# Vacuolar ATPase subunit Atp6v0c transgene promotes neuroprotection and long-distance axon regeneration in injured retinal ganglion neurons

**DOI:** 10.1016/j.omtn.2026.102922

**Published:** 2026-04-01

**Authors:** Anja Kearney, Agnieszka Lukomska, Jacob Brady, Ashiti Damania, Mahit Gupta, Ephraim F. Trakhtenberg

**Affiliations:** 1 Department of Neuroscience, University of Connecticut School of Medicine, 263 Farmington Ave, Farmington, CT 06030, USA

**Keywords:** MT: Oligonucleotides: Therapies and Applications, axon regeneration, optic nerve injury, retinal ganglion cell, gene therapy

## Abstract

Central nervous system (CNS) projection neurons’ failure to repair or regenerate injured axons has devastating consequences for those who have sustained CNS injuries. Thus, there is a need for translatable factors capable of promoting long-distance axon regeneration in the CNS. We hypothesized that supporting lysosomes in injured neurons by supplementing their structural factors through gene therapy may foster axon regeneration. To test our hypothesis, we selected Atp6v0c for experimental regulation because it plays roles in lysosomal acidification and the degradation of misfolded proteins in response to endoplasmic reticulum (ER) stress in injured neurons. We tested this in a rodent optic nerve crush (ONC) model of traumatic optic neuropathy (TON), in which injured prototypical CNS projection neurons, the retinal ganglion cells (RGCs), do not regenerate damaged axons and eventually degenerate. Atp6v0c transgene expression was achieved using intravitreally injected adeno-associated virus serotype 2 (AAV2), which transduces the RGCs. For benchmarking, we compared efficacy to AAV2 targeting of prominent regulators of axon regeneration, Pten, and Klf9. We found that Atp6v0c transgene promoted RGC survival and long-distance axon regeneration, comparable to targeting Pten and Klf9. Thus, Atp6v0c is an axon regeneration-promoting factor with potential for treating CNS injury and disease.

## Graphical abstract

**Figure:**
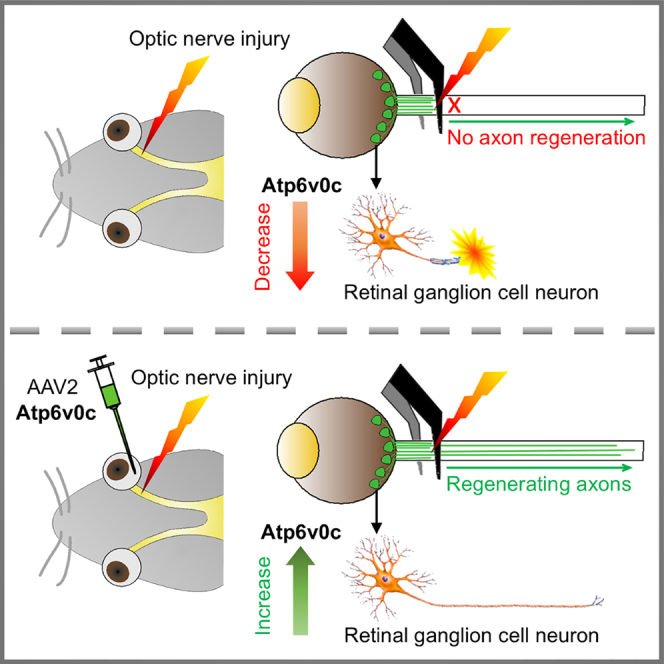

## Teaser

No clinical treatments exist for regenerating damaged axons, limiting recovery options for CNS injuries. Toward addressing this medical need, Trakhtenberg and colleagues, using gene therapy for targeting a transgene to injured neurons, identified the vacuolar acidification enzyme Atp6v0c as an axon regeneration-promoting factor with potential for treating CNS injury and disease.

## Introduction

Central nervous system (CNS) projection neurons’ failure to repair and regenerate injured axons has devastating consequences for those who have sustained spinal cord injury, stroke, brain trauma, or optic neuropathy. Axonal self-repair and regeneration failure in the CNS affects mammals, but not necessarily lower vertebrates, and therefore rodent CNS injury models have been developed to tackle this problem. Like other CNS projection neurons, the retinal ganglion cells (RGCs) do not spontaneously regenerate injured optic nerve axons in an established rodent optic nerve crush (ONC) model of traumatic optic neuropathy (TON).^1^ Oxidative/ endoplasmic reticulum (ER) stress, implicated in neuronal response to injury, is among the earliest pathological events in RGCs after ONC.^2^ Several antioxidant factors are neuroprotective for neuronal survival, but with a few exceptions, they do not generally promote substantial optic nerve axon regeneration *in vivo*. ER stress activates the unfolded protein response, which can be neuroprotective in traumatic/ischemic brain injuries and neurodegenerative conditions.^3^ ER stress also activates lysosomal degradation of misfolded proteins,^4^ and targeting ER-stress-induced lysosomal overload is neuroprotective.^5^ Furthermore, reducing transport of degradative lysosomes into axons leads to degeneration,^6^^,^^7^^,^^8^ while augmenting lysosomal degradation fosters axonal self-repair.^9^^,^^10^ Thus, we hypothesized that supporting lysosomes in injured neurons by supplementing their structural factors through RGC-targeted gene therapy may foster long-distance axon regeneration after ONC *in vivo*. This would imply that a structural factor, established to be critical for lysosomal function, which is dysregulated by axonal injury, might be a viable neurotherapeutic target.

In order to test this implication, we selected Atp6v0c for experimental targeting because (a) it plays roles in lysosomal acidification and degradation of misfolded proteins in response to ER stress^11^ and (b) we found it linked to dysregulation of the lysosome-associated gene network in the injured RGCs (Figure S1A). Atp6v0c is a critical subunit of the vacuolar ATPase (V-ATPase) complex, which is an ATP-driven proton pump that acidifies various vacuolar/vesicular intracellular organelles^12^^,^^13^ and mediates autophagosome/lysosome-mediated degradation of pathogenic proteins in neurodegenerative diseases.^14^ V-ATPase consists of two multi-subunit domains, with the V1 ATPase domain powering the Vo proton-pump domain. The Vo complex itself is comprised of the c-ring core, through which protons are transferred for acidification of the lumen, and supporting subunits (*a*, *d*, *e*, RNAseK, Atp6ap1, Atp6ap2), which interact with the c-core. The c-ring subunits are encoded by two genes, with Atp6v0b contributing 1 *c*” subunit and Atp6v0c contributing 9 identical *c* subunits, with all 10 vertically aligning next to each other in a closed circle, forming a c-ring^15^ (see below). Association of various disorders with deficiencies in Atp6v0c is consistent with this subunit’s major contribution to the critical c-ring. For example, Atp6v0c loss-of-function mutations are embryonically lethal,^16^ while abnormalities in Atp6v0c function are associated with neurodegenerative diseases and lead to dysregulation of autophagy and the lysosomal degradation pathway,^17^ including in Parkinson’s^18^ and Alzheimer’s^19^^,^^20^ diseases. Atp6v0c is also involved in the maintenance of synaptic vesicles,^21^ and its dysregulation results in seizures.^22^ However, it is unknown whether experimental upregulation of Atp6v0c in injured CNS neurons could promote regeneration of damaged axons.

## Results

**Figure 1:**
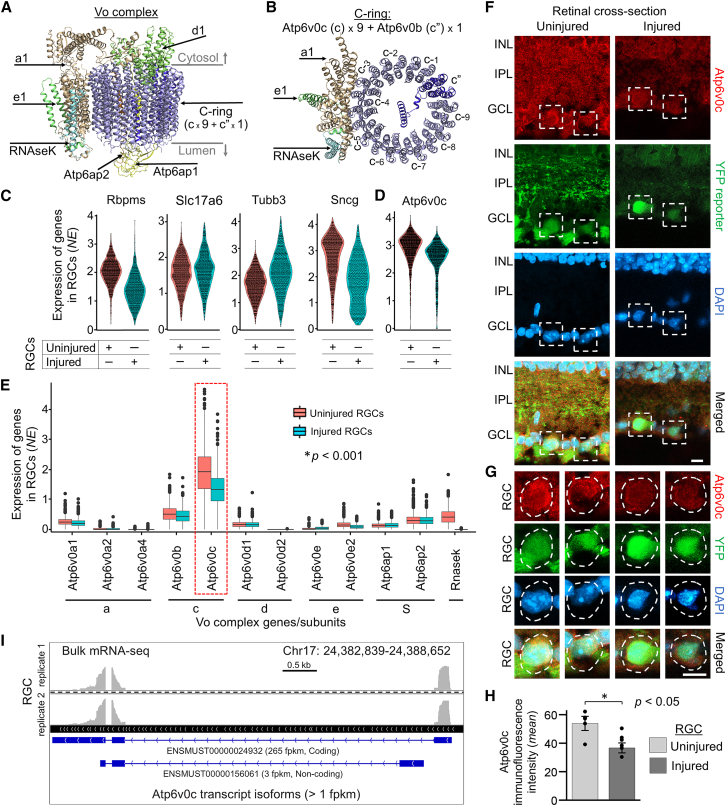
Expression of the V-ATPase Atp6v0c subunit gene is downregulated in RGCs after optic nerve injury (A and B) Schematic of the V-ATPase Vo transmembrane domain, lateral (*A*) and horizontal (*B*) views shown; redrawn from Wang et al. (2020).^15^ Vo complex subunits are indicated, as marked. The c-ring, through which proton is transferred, consists of 9× *c* subunits, encoded by the Atp6v0c gene, and 1× *c*” subunit, encoded by the Atp6v0c gene, as marked (*B*). (C) Gene expression violin plots of pan-RGC marker genes in scRNA-seq-profiled cells, as marked, show that these markers are robustly expressed in both uninjured and injured conditions, confirming RGC cell-type identity. (D) Gene expression violin plot of Atp6v0c in scRNA-seq-profiled RGCs shows that Atp6v0c is downregulated in injured compared to uninjured RGCs. (E) Boxplots showing V-ATPase Vo complex subunit genes expression in scRNA-seq-profiled RGCs before and 2 weeks after ONC injury, as marked. Median ± interquartile range error bars of normalized expression (*NE*) values (not logged) are shown. Statistical significance of differential gene expression was determined by ANOVA (overall *F* = 5317, *p* < 0.001, with *p* values of pairwise comparisons determined by post hoc least significant difference [LSD]). Pairwise differences were significant between uninjured and injured conditions for Atp6v0c (*p* < 0.001), Rnasek (*p* < 0.001), Atp6v0e (*p* < 0.01), and some other genes (not specified due to a small fold change and overall low expression levels); *n* represents individual scRNA-seq-profiled RGCs (*n* = 1683 in uninjured and *n* = 1375 in the injured condition; see materials and methods). (F–H) Confocal microscopy with a 63× objective was used to determine co-localization of Atp6v0c immunofluorescence signal and RGC reporter yellow fluorescent protein (YFP) in the same cells. Representative images of the immunostained retinal cross-sections (*F*), along with higher magnification insets (RGCs outlined with dashed lines; *G*), show reductions in both Atp6v0c granules and diffuse immunofluorescence signal in ONC-injured, compared to uninured, RGCs (in the Thy1-YFP mouse strain, YFP labels the RGCs within the retinal ganglion cell layer; GCL^23^). Apparent reduction of Atp6v0c signal in the inner plexiform layer (IPL) is consistent with degeneration or shrinkage of dead or surviving RGC dendrites and consequent thinning of the IPL between the GCL and the inner nuclear layer (INL) by 2 weeks post-ONC. Scale bars, 10 μm (*F-G*). Atp6v0c immunofluorescence signal intensity was quantified in RGCs (YFP+ labeled cells in the GCL) using ZEN software (Zeiss) measurements tools for average pixel intensity (arbitrary units) and analyzed by independent samples *t* test, 2-tailed; significant difference (*p* < 0.05) indicated by an asterisk ∗. Mean ± SEM shown; *n* = 5–6 cases per group (*H*). (I) Adult uninjured RGC bulk mRNA-seq normalized read alignment to the Atp6v0c gene is shown along with the detected Ensembl transcript isoforms assembled by Cufflinks and normalized by CuffDiff expression values (fpkm), with the protein-coding and non-coding splice variants specified, as marked (see materials and methods). Visualization with IGV viewer.

First, we utilized single cell RNA-seq (scRNA-seq) to characterize whether expression of any of the V-ATPase’s Vo complex subunit members (shown in Figures 1A and 1B) is altered in RGCs after ONC injury. Based on expression of the specific Vo complex genes in scRNA-seq-profiled adult uninjured and 2 weeks post-ONC injured RGCs (cell-type identity confirmed using established pan-RGC markers^24^; Figure 1C), we found that a risk-predisposing change occurred in Atp6v0c expression, as it was significantly downregulated in RGCs after injury (Figures 1D and 1E). While Atp6v0e, encoding subunit *e*, was upregulated after injury, an increase in expression does not pose an apparent loss-of-function risk and may also compensate for downregulation of an alternative *e* subunit isoform, Atp6v0e2, which itself is not high risk, as it is neither ubiquitous nor associated with neurological disorders.^25^ The downregulated RNAseK subunit, however, is another potential target, which we did not pursue here, as it has not been previously associated with any neurological disorders^26^ (Figure 1E). We validated Atp6v0c protein downregulation in the injured RGCs by immunostaining, which showed a significant post-injury decrease (Figures 1F–1H). Considering that Atp6v0c contributes 9 out of 10 c-ring subunits, its downregulation and dysregulation in injured RGCs may hinder self-recovery/repair ability following injury-induced cellular stress, which is consistent with the association between Atp6v0c deficiencies and neurological disorders.^18^^,^^19^^,^^20^^,^^22^ We then used bulk-mRNA-seq-profiling of adult RGCs to identify which Atp6v0c protein-coding transcript isoforms are expressed in RGCs, in order to select an open reading frame (ORF) for experimental transgene vector design (Figure 1I).

**Figure 2:**
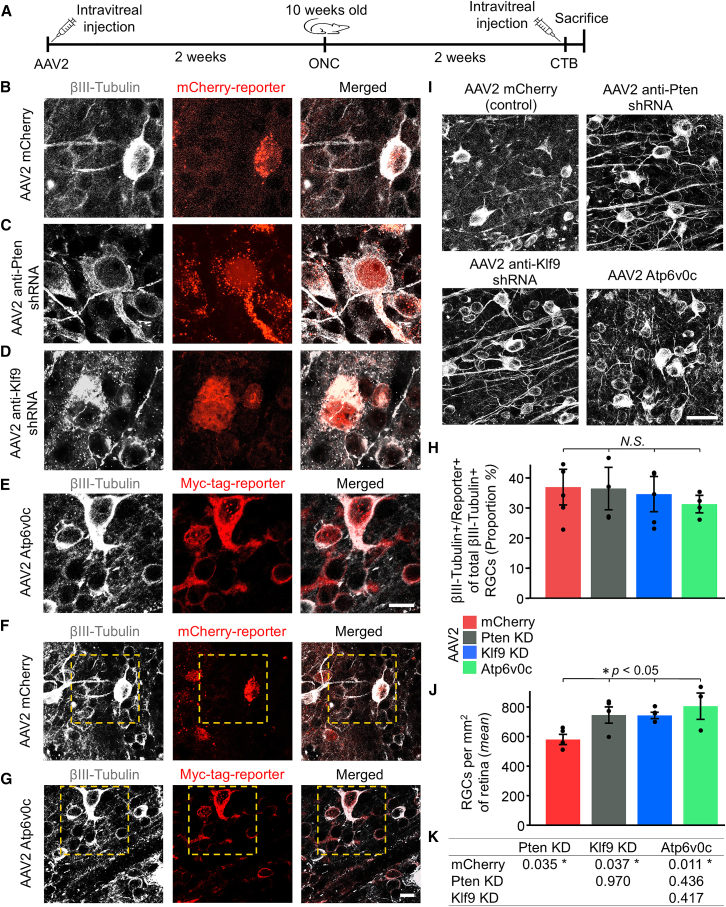
Atp6v0c transgene promotes RGC survival after optic nerve injury (A) Experimental timeline: AAV2 viral vectors were injected intravitreally in 8-week-old mice, and 2 weeks later, ONC injury was performed. Animals were sacrificed for histological analysis 2 weeks after ONC. For axon regeneration studies (shown in Figure 3), the axonal tracer CTB was injected intravitreally 1 day prior to sacrifice. (B–E) Confocal microscopy with a 63× objective was used to determine co-localization of vector reporters and RGC marker in the same cells. High-magnification representative images of the immunostained retinal flat mounts’ GCL show that mCherry and Myc-tag reporters of transduction/expression localize to βIII-Tubulin+ cells (βIII-Tubulin is a neuronal-specific marker that labels the RGCs within the retina), and that transduced cells are detected at similar proportions in all conditions, as marked (*B-E*). Scale bars, 10 μm. (F and G) Lower-magnification, larger field-of-view representative images for the control with mCherry reporter (*F*; dashed-line outlined inset shown in *B*) and for the Atp6v0c transgene with mCherry Myc-tag reporter (*G*; dashed-line outlined inset shown in *E*) vectors, processed as above. Scale bars, 10 μm. (H) Proportion of βIII-Tubulin+/Reporter+ cells among total βIII-Tubulin+ RGCs (*%*) in the GCL, analyzed by ANOVA with post hoc LSD. No significant differences were found. Mean ± SEM shown; *n* = 4 cases per group. *N.S.* = not significant. (I) Representative images of retinal flat mounts’ GCL immunostained for an RGC marker βIII-Tubulin at 2 weeks after ONC, pre-treated with experimental and control AAV2 vectors, as marked. Sale bars, 20 μm. (J and K) Quantitation of RGC (βIII-Tubulin+ cells) survival in retinal flat mounts’ GCL at 2 weeks after ONC, from the experimental and control treatment conditions, as marked. Mean ± SEM) shown; *n* = 3–4 cases per group (*J*). Data analyzed using ANOVA, overall *F* = 3.68, *p* < 0.05, with *p* values of pairwise comparisons determined by post hoc LSD; significant differences (*p* < 0.05) indicated by an asterisk ∗ (*K*).

Next, we tested whether sustained upregulation of Atp6v0c in injured RGCs is sufficient to promote axon regeneration after ONC. For experimental Atp6v0c upregulation, we selected from the transcript-specific bulk-mRNA-seq-profiled RGC dataset an ORF of the only detected protein-coding Atp6v0c transcript identified in both replicates (Figure 1I). Stable experimental neuronal Atp6v0c transgene expression was achieved using intravitreally injected adeno-associated virus serotype 2 (AAV2), which transduces the RGCs. For benchmarking, we compared the effects to AAV2 targeting of prominent regulators of long-distance axon regeneration, Pten, and Klf9.^24^^,^^27^^,^^28^ To validate AAV2 vector expression (which transduces RGCs within the retina) in injured RGCs, a Myc reporter was added to the *N*-terminus of the Atp6v0c ORF. For negative control, an mCherry-expressing AAV2 vector was used. For positive control, established axon regeneration-promoting AAV2 vectors expressing short hairpin RNAs (shRNAs) to knock down (KD) expression of Pten or Klf9, and co-expressing an mCherry reporter, were used.^24^^,^^28^ The viruses were injected intravitreally in adult mice, and 2 weeks later, ONC injury was performed (Figure 2A). Co-immunostaining of the retinas 2 weeks after ONC for the Myc-tag reporter and βIII-Tubulin (a neuronal-specific marker that selectively labels the RGCs within the retina) confirmed expression of the Atp6v0c transgene in the transduced (Myc reporter-labeled) injured RGCs, with transduction efficiency of ∼30%, similar to prior reports that also used AAV2 to target the RGCs^24^ (as shown by confocal images in Figures 2B–2E, wider field-of-view images in Figures 2F and 2G, and quantifications in Figure 2H); transgene expression was also validated by scRNA-seq (Figure S1B). mCherry reporter-labeled injured RGCs also confirmed comparable transduction and expression of the established AAV2 vectors used here for negative (mCherry) and positive (anti-Pten and anti-Klf9 shRNAs) controls (Figures 2B–2D and 2H).

**Figure 3:**
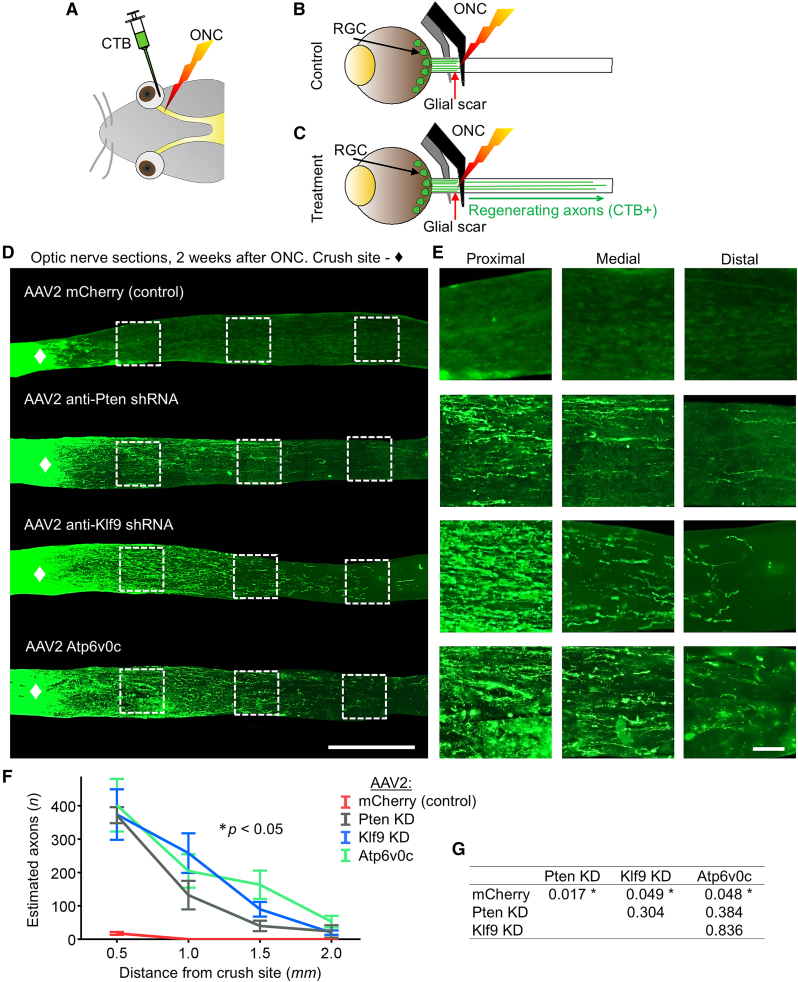
Atp6v0c transgene promotes axon regeneration after optic nerve injury (A–C) Experimental timeline shown in Figure 2A. For visualization of the regenerating axons, or their absence, an axonal tracer CTB (conjugated to Alexa Fluor 488 dye) is injected intravitreally 1 day before sacrifice. It is rapidly taken by the RGCs and transported anterogradely along the intact axons that are connected to RGC soma (*A*). In the control condition, the CTB-labeled axons end abruptly at the ONC injury site (*B*), but in the experimental treatment condition, the regenerating axons are CTB-labeled also beyond the injury site, to the length they regrew along the optic nerve in 2 weeks (a standard endpoint in axon regeneration proof-of-concept studies) (*C*). (D and E) Representative images of optic nerve longitudinal cryosections with CTB-labeled axons at 2 weeks after ONC from animals pre-treated with experimental and control AAV2 vectors, as marked (*D*). Insets: Representative images of the optic nerve regions proximal and distal to the injury site are magnified for better visualization of the axons or their absence (*E*). The edges of the tissue were optically trimmed (i.e., cropped) due to artifactual autofluorescence that is common at tissue edges (see materials and methods). Scale bars, 500 μm (main panels), 50 μm (insets). (F and G) Quantitation of CTB-labeled regenerating axons at 2 weeks after ONC, at increasing distances from the injury site, after pre-treatment with experimental and control AAV2 vectors, as marked. Mean ± SEM shown; *n* = 3–4 cases per group (*F*). Data analyzed using repeated-measures ANOVA with post hoc LSD, sphericity assumed, overall *F* = 6.28, *p* < 0.05; post hoc *p* value (*p* < 0.05) indicated by an asterisk ∗ (*G*).

Then, we tested whether directly upregulating Atp6v0c in injured RGCs would promote RGC survival and axon regeneration after injury. We used an established 2-week post-ONC axon regeneration assay,^24^^,^^27^^,^^28^ in which the optic nerve is injured by crush in order to sever all RGC axons, and RGC survival and axon regeneration are assayed at 2 weeks after injury. AAV2 vectors expressing Atp6v0c, anti-Pten shRNA, anti-Klf9 shRNA, or mCherry control were injected intravitreally in adult mice, and 2 weeks later, ONC was performed. To visualize the regenerating axons or their absence, the axonal tracer Cholera toxin subunit B (CTB)-488 was injected 1 day prior to sacrifice at 2 weeks after ONC. The number of regenerating axons was quantified in longitudinal sections of the optic nerve (no spared axons were detected in either group), and RGC survival was quantified in retinal flat mounts (see materials and methods for details; experimental timeline in Figure 2A). We found that Atp6v0c upregulation promoted RGC survival by approximately 25%, which is a similar extent of neuroprotection as with anti-Pten shRNA and anti-Klf9 shRNA AAV2 treatments by 2 weeks post-ONC, compared to RGC survival in the injured control group (Figures 2I–2K). Although, given that over 80% of RGCs die by 2 weeks after ONC,^29^ the observed increase in survival is modest (as only about 5% fewer RGCs died), if experimental neuroprotection persists permanently, this would be meaningful because, without treatment, most or nearly all RGCs eventually die. Importantly, relative to only minor axonal sprouting past the injury site detected in control animals (as expected), the Atp6v0c transgene promoted long-distance axon regeneration past the injury site, which is comparable to the extent of axon regeneration achieved by anti-Pten shRNA and anti-Klf9 shRNA AAV2 treatments (Figures 3A–3G).

## Discussion

No clinical treatments exist for regenerating damaged axons, limiting recovery options for CNS injuries. Toward addressing this unmet medical need, using gene therapy for targeting a transgene to injured neurons, we identified the V-ATPase subunit Atp6v0c as an axon regeneration-promoting factor with neurotherapeutic potential for CNS injury and disease. Remarkably, the extent of neuroprotection and axon regeneration was comparable to the effects of AAV2 targeting Pten and Klf9, which are benchmark regulators of CNS axon regeneration.^24^^,^^27^^,^^28^ Although conditional genetic knockout of Pten leads to greater survival and axon regeneration than AAV2-mediated KD,^27^ we used the same viral vector delivery method in all conditions for a more appropriate comparison of the treatments *per se*, which better reflects their translational potential via gene therapy.

The limitations and future directions are as follows. For a proof of concept, we used the standard in the field 2-week post-ONC time point to assess whether the Atp6v0c transgene has neurotherapeutic potential. Future long-term studies are needed to explore the full potential, as prior studies suggested that ∼3 months are needed post-ONC for experimentality stimulated regenerating axons to reach post-synaptic targets in respective brain nuclei for recovery of visual functions.^30^^,^^31^ Future long-term studies should also examine Atp6v0c levels at additional time points post-injury and explore whether treatment effects vary across RGC subtypes or retinal regions. Additionally, leveraging emerging improved AAV2-based vectors with higher transduction efficiency could further enhance therapeutic efficacy. Furthermore, as a greater extent of axon regeneration is achievable by combining different approaches,^30^^,^^31^ future studies may find that Atp6v0c upregulation cooperates with other treatments to yield an even greater extent of axonal repair/regeneration. A number of effectors may be implicated in the neurotherapeutic efficacy we observed. For example, enhancing lysosomal function in injured neurons may promote axon regeneration by facilitating a higher demand for lysosomal degradation of proteins that misfolded either due to ER stress or an increased protein synthesis demand for supporting axonal regeneration in a pathological microenvironment. This could be particularly relevant for the degradative lysosomes transported for function into damaged axons. Exploring these possibilities, along with the upstream and downstream molecular mechanisms, represents an important future research direction. For example, future studies could explore whether the Atp6v0c transgene rescues a specific lysosomal dysfunction by mitigating ER stress, regulating autophagy, and/or improving lysosomal acidification and clearing misfolded proteins. Moreover, because previous association of Atp6v0c with epilepsy and neurodevelopmental disorders was due to congenital mutations in the Atp6v0c gene,^22^ upregulating wild-type Atp6v0c is not apparently concerning; nevertheless, future translational studies would need to evaluate the safety of increasing Atp6v0c levels in injured neurons.

## Materials and methods

Materials And Methods are provided in the supplemental information.

## Data and code availability

• The plasmid for the Atp6v0c AAV2 vector will be shared for academic purposes. This study did not generate any other new unique reagents.
• RNA sequencing data from this study have been deposited in the Gene Expression Omnibus (GEO) at the NCBI repository and are publicly available (accession number GSE325128).
• This paper does not report original code.
• Any additional information required to reanalyze the data reported in this paper is available from the lead contact upon request.

## Acknowledgments

This work was supported by a grant from the 10.13039/100000002National Institutes of Health (NIH), National Eye Institute (NEI) (grant R01-EY029739, to E.F.T.). Portions of this research were conducted at the High Performance Computing Facility, University of Connecticut. We are grateful to Bill Flynn and Elise Courtois (The Jackson Laboratory for Genomic Medicine, Farmington, CT) for single-cell RNA-sequencing service and to Sophan Iv (Research IT Services, University of Connecticut) and Stephen King (High Performance Computing Facility, University of Connecticut) for bioinformatics support. We thank Mathew Frost, Madison Sakheim, and Lucy Homer (students, University of Connecticut School of Medicine) for technical assistance.

## Author contributions

A.K.^#^, A.L.^#^, J.B., A.D., and M.G., performed the experiments. E.F.T. designed the study and wrote the manuscript.

## Declaration of interests

The authors declare no competing interests.

## Supplemental information

Document S1. Figure S1

Document S2. Article plus supplemental information

## References

[1] Ghaffarieh A., Levin L.A. (2012). Optic nerve disease and axon pathophysiology. Int. Rev. Neurobiol..

[2] Kanamori A., Catrinescu M.M., Kanamori N., Mears K.A., Beaubien R., Levin L.A. (2010). Superoxide is an associated signal for apoptosis in axonal injury. Brain.

[3] Hetz C., Saxena S. (2017). ER stress and the unfolded protein response in neurodegeneration. Nat. Rev. Neurol..

[4] Reggiori F., Molinari M. (2022). ER-phagy: mechanisms, regulation, and diseases connected to the lysosomal clearance of the endoplasmic reticulum. Physiol. Rev..

[5] Bisicchia E., Mastrantonio R., Nobili A., Palazzo C., La Barbera L., Latini L., Millozzi F., Sasso V., Palacios D., D'Amelio M., Viscomi M.T. (2022). Restoration of ER proteostasis attenuates remote apoptotic cell death after spinal cord injury by reducing autophagosome overload. Cell Death Dis..

[6] Farfel-Becker T., Roney J.C., Cheng X.T., Li S., Cuddy S.R., Sheng Z.H. (2019). Neuronal Soma-Derived Degradative Lysosomes Are Continuously Delivered to Distal Axons to Maintain Local Degradation Capacity. Cell Rep..

[7] Roney J.C., Cheng X.T., Sheng Z.H. (2022). Neuronal endolysosomal transport and lysosomal functionality in maintaining axonostasis. J. Cell Biol..

[8] Maday S. (2016). Mechanisms of neuronal homeostasis: Autophagy in the axon. Brain Res..

[9] He M., Ding Y., Chu C., Tang J., Xiao Q., Luo Z.G. (2016). Autophagy induction stabilizes microtubules and promotes axon regeneration after spinal cord injury. Proc. Natl. Acad. Sci. USA.

[10] Ko S.H., Apple E.C., Liu Z., Chen L. (2020). Age-dependent autophagy induction after injury promotes axon regeneration by limiting NOTCH. Autophagy.

[11] Sun Y., Wang X., Yang X., Wang L., Ding J., Wang C.C., Zhang H., Wang X. (2023). V-ATPase recruitment to ER exit sites switches COPII-mediated transport to lysosomal degradation. Dev. Cell.

[12] Forgac M. (2007). Vacuolar ATPases: rotary proton pumps in physiology and pathophysiology. Nat. Rev. Mol. Cell Biol..

[13] Zhao J., Benlekbir S., Rubinstein J.L. (2015). Electron cryomicroscopy observation of rotational states in a eukaryotic V-ATPase. Nature.

[14] Lee J.H., Yu W.H., Kumar A., Lee S., Mohan P.S., Peterhoff C.M., Wolfe D.M., Martinez-Vicente M., Massey A.C., Sovak G. (2010). Lysosomal proteolysis and autophagy require presenilin 1 and are disrupted by Alzheimer-related PS1 mutations. Cell.

[15] Wang L., Wu D., Robinson C.V., Wu H., Fu T.M. (2020). Structures of a Complete Human V-ATPase Reveal Mechanisms of Its Assembly. Mol. Cell.

[16] Aoto K., Kato M., Akita T., Nakashima M., Mutoh H., Akasaka N., Tohyama J., Nomura Y., Hoshino K., Ago Y. (2021). ATP6V0A1 encoding the a1-subunit of the V0 domain of vacuolar H. Nat. Commun..

[17] Mangieri L.R., Mader B.J., Thomas C.E., Taylor C.A., Luker A.M., Tse T.E., Huisingh C., Shacka J.J. (2014). ATP6V0C knockdown in neuroblastoma cells alters autophagy-lysosome pathway function and metabolism of proteins that accumulate in neurodegenerative disease. PLoS One.

[18] George J., Shafiq K., Kapadia M., Kalia L.V., Kalia S.K. (2024). High frequency electrical stimulation reduces α-synuclein levels and α-synuclein-mediated autophagy dysfunction. Sci. Rep..

[19] Liu Q.Y., Lei J.X., Sikorska M., Liu R. (2008). A novel brain-enriched E3 ubiquitin ligase RNF182 is up regulated in the brains of Alzheimer's patients and targets ATP6V0C for degradation. Mol. Neurodegener..

[20] Kim S.H., Cho Y.S., Kim Y., Park J., Yoo S.M., Gwak J., Kim Y., Gwon Y., Kam T.I., Jung Y.K. (2023). Endolysosomal impairment by binding of amyloid beta or MAPT/Tau to V-ATPase and rescue via the HYAL-CD44 axis in Alzheimer disease. Autophagy.

[21] Abbas Y.M., Wu D., Bueler S.A., Robinson C.V., Rubinstein J.L. (2020). Structure of V-ATPase from the mammalian brain. Science.

[22] Mattison K.A., Tossing G., Mulroe F., Simmons C., Butler K.M., Schreiber A., Alsadah A., Neilson D.E., Naess K., Wedell A. (2023). ATP6V0C variants impair V-ATPase function causing a neurodevelopmental disorder often associated with epilepsy. Brain.

[23] Hass D.T., Barnstable C.J. (2019). Mitochondrial Uncoupling Protein 2 Knock-out Promotes Mitophagy to Decrease Retinal Ganglion Cell Death in a Mouse Model of Glaucoma. J. Neurosci..

[24] Rheaume B.A., Xing J., Lukomska A., Theune W.C., Damania A., Sjogren G., Trakhtenberg E.F. (2023). Pten inhibition dedifferentiates long-distance axon-regenerating intrinsically photosensitive retinal ganglion cells and upregulates mitochondria-associated Dynlt1a and Lars2. Development.

[25] Blake-Palmer K.G., Su Y., Smith A.N., Karet F.E. (2007). Molecular cloning and characterization of a novel form of the human vacuolar H+-ATPase e-subunit: an essential proton pump component. Gene.

[26] Makar A.N., Boraman A., Mosen P., Simpson J.E., Marques J., Michelberger T., Aitken S., Wheeler A.P., Winter D., von Kriegsheim A., Gammoh N. (2024). The V-ATPase complex component RNAseK is required for lysosomal hydrolase delivery and autophagosome degradation. Nat. Commun..

[27] Park K.K., Liu K., Hu Y., Smith P.D., Wang C., Cai B., Xu B., Connolly L., Kramvis I., Sahin M., He Z. (2008). Promoting axon regeneration in the adult CNS by modulation of the PTEN/mTOR pathway. Science.

[28] Apara A., Galvao J., Wang Y., Blackmore M., Trillo A., Iwao K., Brown D.P., Fernandes K.A., Huang A., Nguyen T. (2017). KLF9 and JNK3 Interact to Suppress Axon Regeneration in the Adult CNS. J. Neurosci..

[29] Mead B., Tomarev S. (2016). Evaluating retinal ganglion cell loss and dysfunction. Exp. Eye Res..

[30] de Lima S., Koriyama Y., Kurimoto T., Oliveira J.T., Yin Y., Li Y., Gilbert H.Y., Fagiolini M., Martinez A.M.B., Benowitz L. (2012). Full-length axon regeneration in the adult mouse optic nerve and partial recovery of simple visual behaviors. Proc. Natl. Acad. Sci. USA.

[31] Lim J.H.A., Stafford B.K., Nguyen P.L., Lien B.V., Wang C., Zukor K., He Z., Huberman A.D. (2016). Neural activity promotes long-distance, target-specific regeneration of adult retinal axons. Nat. Neurosci..

