# Supplementary material for: Vacuolar ATPase subunit Atp6v0c transgene promotes neuroprotection and long-distance axon regeneration in injured retinal ganglion neurons
Source: Mol Ther Nucleic Acids. 2026 Apr 1;37(2):102922. doi: 10.1016/j.omtn.2026.102922 (PMC13096995; doi:10.1016/j.omtn.2026.102922)
Supplement: Document S1. Figure S1 [file mmc1.pdf]

OMTN, Volume *37*

Supplemental information

Vacuolar ATPase subunit Atp6v0c transgene promotes neuroprotection and long-distance axon regeneration in injured retinal ganglion neurons

Anja Kearney, Agnieszka Lukomska, Jacob Brady, Ashiti Damania, Mahit Gupta, and Ephraim F. Trakhtenberg

MATERIALS AND METHODS

Animal use, surgeries, intraocular injections. All animal studies were performed at the University of Connecticut Health Center with approval of the Institutional Animal Care and Use Committee and of the Institutional Biosafety Committee, and performed in accordance with the ARVO Statement for the Use of Animals in Ophthalmic and Visual Research. Mice were housed in the animal facility with a 12-h light/12-h dark cycle (lights on from 7:00 AM to 7:00 PM) and a maximum of five adult mice per cage. Food and water were available ad libitum. The study used wild-type 129S1/SvImJ (JAX strain 002448) mice, as well as Thy1-YFP reporter mice (which also independently co-express Cre^{ERT2}; JAX strain 012708) transferred to 129S1/SvImJ background. In these transgenic reporter mice, within the retina, YFP labels the RGCs[1]. Optic nerve surgeries and intravitreal injections, were carried out on mice of both sexes 8-12 weeks of age (average body weight 20-26 g) under general anesthesia, as described previously[2,3]. For histological analysis, mice were euthanized using CO2 and cervical dislocation. The viruses included AAV2 vectors expressing Atp6v0c (ORF of ENSMUST00000024932), anti-Pten shRNAs (target sequences: 5′-GCAGAAACAAAAGGAGATATCA-3′, 5′-GATGATGTTTGAAACTATTCCA-3′, 5′-GTAGAGTTCTTCCACAAACAGA-3′, and 5′-GATGAAGATCAGCATTCACAAA-3′), anti-Klf9 shRNAs (target sequences: 5′-GGAGGCGCTGCCGGTTACGTA-3′, 5′-TGGCTGCCCAGTGTCTGGTTT-3′, 5′-CGGGGGACACCTGGAAGGATT-3′, and 5′-GCAAATAAATGCTTTTGGTAC-3′), and mCherry alone for control (titers ~1×10^{12} GC/mL; VectorBuilder, Inc.). Vectors expressing anti-Pten or anti-Klf9 shRNAs, also co-expressed an mCherry reporter. Atp6v0c and mCherry ORFs had an *N*-terminally fused myc-tag reporter. Viruses (2 µl per eye) were injected intravitreally, avoiding injury to the lens, in 8-week-old mice, which were randomly assigned to experimental or control conditions, 2 weeks prior to ONC surgery. This lead time allowed for sufficient transduction and expression of the transgenes in RGCs at the time of ONC. Transduction efficiency was approximately 30%, which is comparable to prior reports that also used AAV2 to target the RGCs[3]. Cholera toxin subunit B (CTB) conjugated to Alexa Fluor 488 dye (C34775, ThermoFisher Scientific) was injected (1% in 3 µl PBS) intravitreally one day prior to sacrifice, at 2 weeks after ONC, in order to visualize the regenerating axons or their absence.

Tissue processing and immunostaining. Standard histological procedures were used, as described previously[2,3]. Briefly, anesthetized mice were transcardially perfused with isotonic saline followed by 4% paraformaldehyde (PFA), the eyes and the optic nerves were dissected, the cornea was punctured, and the tissues were postfixed 2 hours. The retinas dissected-out for flat-mounts or horizontal flat-sections, the whole eyes for sagittal cross-sections, and the optic nerves for longitudinal sections, were washed in PBS and transferred to 30% sucrose overnight at 4 °C. The optic nerves were then embedded in OCT Tissue Tek Medium (Sakura Finetek), frozen, cryosectioned longitudinally at 14 μm, and then mounted for imaging on coated glass slides. For analyzing RGC survival, resected (into PBS at 4 °C) free-floating retinas were immunostained in 24-well plate wells and, after making 4 symmetrical slits, flat-mounted on coated glass slides for imaging. For analyzing Atp6v0c transgene expression in AAV2-transduced RGCs, the flattened retinas were embedded in OCT Tissue Tek Medium (Sakura Finetek), frozen, and cryosectioned at 14 μm horizontality to capture the ganglion cell layer (GCL) of the retina, and then immunostained and mounted on coated glass slides for imaging using a confocal microscope (see below). For Atp6v0c immunostaining, the whole eyes were embedded in OCT Tissue Tek Medium (Sakura Finetek), frozen, and cryosectioned at 14 μm sagittally (capturing the GCL in the cross-sections), and then immunostained and mounted on coated glass slides for imaging using a confocal microscope (see below). For immunostaining, the tissues were blocked with appropriate sera, incubated overnight at 4 °C with primary antibodies, Atp6v0c (1:100; rabbit polyclonal, PA5116676 Thermo Fisher Scientific), βIII-Tubulin (1:500; rabbit polyclonal, Ab18207 Abcam), and Myc (1:400; mouse monoclonal, SC-40 SCBT), then washed 3 times, incubated with appropriate fluorescent dye-conjugated secondary antibodies (1:500; IgG H+L or IgG 2a Alexa Fluor, Thermo Fisher Scientific) overnight at 4 °C, washed 3 times again, and mounted for imaging.

Quantification of regenerated axons and RGC survival. To visualize the regenerating axons or their absence after treatments, axonal tracer (Alexa Fluor 488-conjugated CTB 1% in 3 μl PBS) was intravitreally injected one day before animals were euthanized 2 weeks following ONC. Longitudinal sections of the optic nerve were examined for possible axon sparing. No spared axons were found in control, and no evidence of axon sparing was found in experimental conditions (i.e., at 2 weeks after injury, no axons were found at the most distal from the injury region of the optic nerve). Regenerated axons (defined as continuous fibers, which are absent in

controls and are discernible from background puncta and artefactual structures) were counted manually using a fluorescent microscope (40x/1.2 C-Apochromat W; AxioObserver.Z1, Zeiss) in at least 4 longitudinal sections per optic nerve at various distances from the injury site (identified by the abrupt disruption of axonal density approximately 1 mm from the optic nerve head, as marked by a rhombus in Fig. 3), and these values were used to estimate the total number of regenerating axons per nerve, as described[2,3]. For representative images, serial fields of view along the longitudinal optic nerve tissue section were imaged as above; *z*-stacks with 5 planes at 0.5 μm intervals were deconvoluted, merged, and stitched (ZEN software, Zeiss). Then, processed images of 3 tissue sections from the same optic nerve were superimposed over each other and merged using Photoshop CS6 (Adobe), shown as representative images. RGC survival was quantified in retinal flatmounts' GCL as described[2,3], by immunostaining with an antibody to βIII-Tubulin (neuronal marker), taking advantage of the selective expression (within the retina) of βIII-tubulin in RGCs. ImageJ software Cell Counter Plugin was used to count βIII-Tubulin positive cells from images acquired (using a fluorescent microscope, 20x LD; Zeiss, AxioObserver.Z1, Zeiss) at 1 mm and at 2 mm from the optic nerve head in four directions of the GCL, then averaged to estimate overall RGC survival per mm^2 of the retina. For analyzing RGC transduction efficiency, the horizontality cryosectioned flattened retinas' GCL immunostained for βIII-Tubulin and Myc reporter were imaged along with mCherry reporter (where applicable) using confocal microscopy (63x Oil; LSM800, Zeiss), and proportions of βIII-Tubulin+/reporter+ of total βIII-Tubulin+ RGCs were quantified; representative images were sampled randomly. For quantifications and representative images of Atp6v0c immunostained RGCs, Thy1-YFP mice's retinal GCL regions randomly sampled from the sagittally cryosectioned retinas' immunostained for Atp6v0c were imaged, along with the YFP RGC marker, using a confocal microscope (63x objective; LSM800, Zeiss). Atp6v0c immunofluorescence signal intensity was measured in the YFP+ RGCs located in the GCL, using ZEN software (Zeiss) measurements tools for average pixel intensity. Quantifications were performed on individual RGCs present in the 63x image field of view GCL, in 4 eye/retinal cross-sections per condition. Investigators performing the surgeries and quantifications were masked to the group identity by another researcher until the end of the experiment.

Statistical analyses. All tissue processing, quantification, and data analysis were done masked throughout the study. Sample sizes were based on accepted standards in the literature and our prior experiences. Sample size

represents total number of biological replicates in each condition. All experiments included appropriate controls. No cases were excluded in our data analysis, although a few animals that developed a cataract in the injured eye were excluded from the study, and their tissues were not processed. The data are presented as means ± SEM, and was analyzed (as specified in the applicable Figure legends) by independent samples *t*-test (2-tailed) or by ANOVA with or without Repeated Measures and a posthoc LSD test (SPSS). All differences were considered significant at $p < 0.05$.

RGC scRNA-seq and bulk-mRNA-seq datasets. ScRNA-seq was performed as we described previously[3], briefly: uninjured and injured (2 weeks post-ONC) RGCs from 12-week old mice of both sexes were Thy1-immunopanned from single cell retinal suspension (10 retinas per batch), after immunopanning depletion of macrophages and amacrine cells. Cells were resuspended in DPBS with 0.04% BSA, and immediately processed as follows. Cell count and viability were determined using trypan blue on a Countess FL II, and 6,000 cells per batch were loaded for capture onto the Chromium System using the v2 single cell reagent kit (10X Genomics). Following capture and lysis, cDNA was synthesized and amplified (12 cycles) as per manufacturer's protocol (10X Genomics). The amplified cDNA from each channel of the Chromium System was used to construct an Illumina sequencing library and sequenced on HiSeq 4000 with 150 cycle sequencing. Illumina basecall files (*.bcl) were converted to FASTQs using CellRanger v1.3, which uses bcl2fastq v2.17.1.14. FASTQ files were then aligned to mm39 mouse reference genome and transcriptome using the CellRanger v1.3 software pipeline with default parameters, which demultiplexes the samples and generates a gene versus cell expression matrix based on the barcodes and assigned unique molecular identifiers (UMIs) that enable determining the individual cells from which each RNA molecule originated. For determining gene expression, normalization of the raw counts was performed using Seurat v5.2.1's NormalizeData function, which divides the feature counts by the number of counts per each cell and then applies natural log transformation, resulting in normalized expression (*NE*) values. Individual samples were batch adjusted and integrated using Seurat's FindVariableFeatures, SelectIntegrationFeatures, FindIntegrationAnchors, and IntegratedData functions. RGC cell identity was confirmed based on co-expression of pan-RGC markers (Rbpms, Slc17a6, Sncg, and Tubb3). A total of 1683 RGCs from uninjured and 1375 RGCs from injured retinas that passed quality control (QC) were selected. QC filters/thresholds included the following criteria per cell: a maximum threshold of 20% mitochondrial genes

expressed in the transcriptome, a minimum of 500 genes, and a maximum of 150,000 UMIs (to mitigate the presence of cell doublets). Uninjured and injured datasets were then merged and normalized using Seurat's merge and NormalizeData functions. Violin plots of normalized gene expression were generated using Seurat's VlnPlot function, and overlayed categorical scatter (violin point) plots were generated using ggbeeswarm. Boxplots for median relative counts of normalized expression (not-logged, with median ± interquartile range error bars) of V-ATPase Vo subunit genes in the uninjured and injured RGCs were generated using Seurat's NormalizeData function (with Relative Counts normalization and a scaling factor of 1000) and visualized using ggplot2. For bulk-mRNA-seq, adult uninjured RGCs transduced with AAV2 (~30% transduction efficiency) expressing mCherry were Thy1-immunopanned from single cell retinal suspension (from 10 retinas after immunopanning depletion of macrophages) and FACS'ed for mCherry+ cells (as we described previously[3]). Approximately, 5,000 RGCs were collected by FACS and RNA was isolated immediately using the Zymo QuickRNA microprep kit. Total RNA with RNA Integrity Number (RIN) ≥ 9 (by Bioanalyzer 2100 using the Nano 6000 kit, Agilent) was extracted using Direct-zol RNA MiniPrep kit (R2050, Zymo Research). cDNA libraries were prepared using polyA-selected RNA (TruSeq RNA Library Prep Kit, Illumina). Paired reads were sequenced in a DNA-strand-specific manner, 100 bp from each end on HiSeq 2000 Sequencer (Illumina), passed QC filters, mapped to the mm39 genome and transcriptome by Hisat2, and gene expression was analyzed by Cufflinks (following a pipeline that we previously published). Transcript isoforms expressed > 1 FPKM in both replicates were visualized using IGV browser.

Data availability. The scRNA-seq dataset from adult uninjured and injured RGCs that we generated for these studies is available through the NCBI GEO accession GSE325128. Adult uninjured RGC bulk-mRNA-seq raw reads and processed data for expression of transcripts isoforms from the Atp6v0c gene locus analyzed in this study are available through the NCBI GEO under accession number GSE252517 (which we generated previously).

METHODS FOR FIGURE S1

Gene-concept network plot analysis was performed using the methods we described previously[3-5]. Briefly, genes differentially expressed (logfc.threshold = 0.4, min.pct = 0.5) in adult uninjured vs injured RGCs were identified using Seurat's FindMarkers function[6,7]. Those expressed > 0.1 *NE* in the higher expressing condition were analyzed using the R package clusterProfiler, using all genes expressed in injured RGCs as background (*NE* > 0). False discovery rate (FDR) was used for multiple testing correction, and a minimum *p*-value of 0.05 was set as cutoff for significance of GO terms enrichment. GO:BP terms were rank ordered by decreasing significance (FDR adjusted *p*-value). Fold-enrichment was calculated as the DEG ratio divided by the background ratio[8]. Significantly enriched GO:BP pathways (*p*-value < 0.05) containing Atp6v0c gene were plotted in a Gene-Concept Network Plot using the clusterProfiler and enrichplot R packages[9].

Validation of Atp6v0c transgene expression by scRNA-seq. RGC scRNA-seq is detailed in the main text Materials And Methods, and was performed similarly on the RGCs treated with AAV2-Atp6v0c. Briefly, at 2 weeks post-ONC, CTB-488 stereotaxically injected into the distal optic nerve uptaken by the regenerated axons and retrogradely transported to the soma of RGCs in the retina. The next day, the responding RGCs FACS-isolated and processed by scRNA-seq, as described in the main text Materials And Methods, with the following additional steps. AAV2 vector transgene with UTRs was custom-added to the reference genome and transcriptome GTF. Reads mapping to the AAV2 vector transgene UTRs were detected only in the AAV2-Atp6v0c-treated condition, confirming transgene expression in the responding RGCs. For determining Atp6v0c gene *NE*, counts of the reads that mapped non-redundantly (i.e., reads that mapped to both were counted only ones) to the endogenous and transgene Atp6v0c were combined. More detailed methods for these procedures are described in our previous publications[3,10-12].

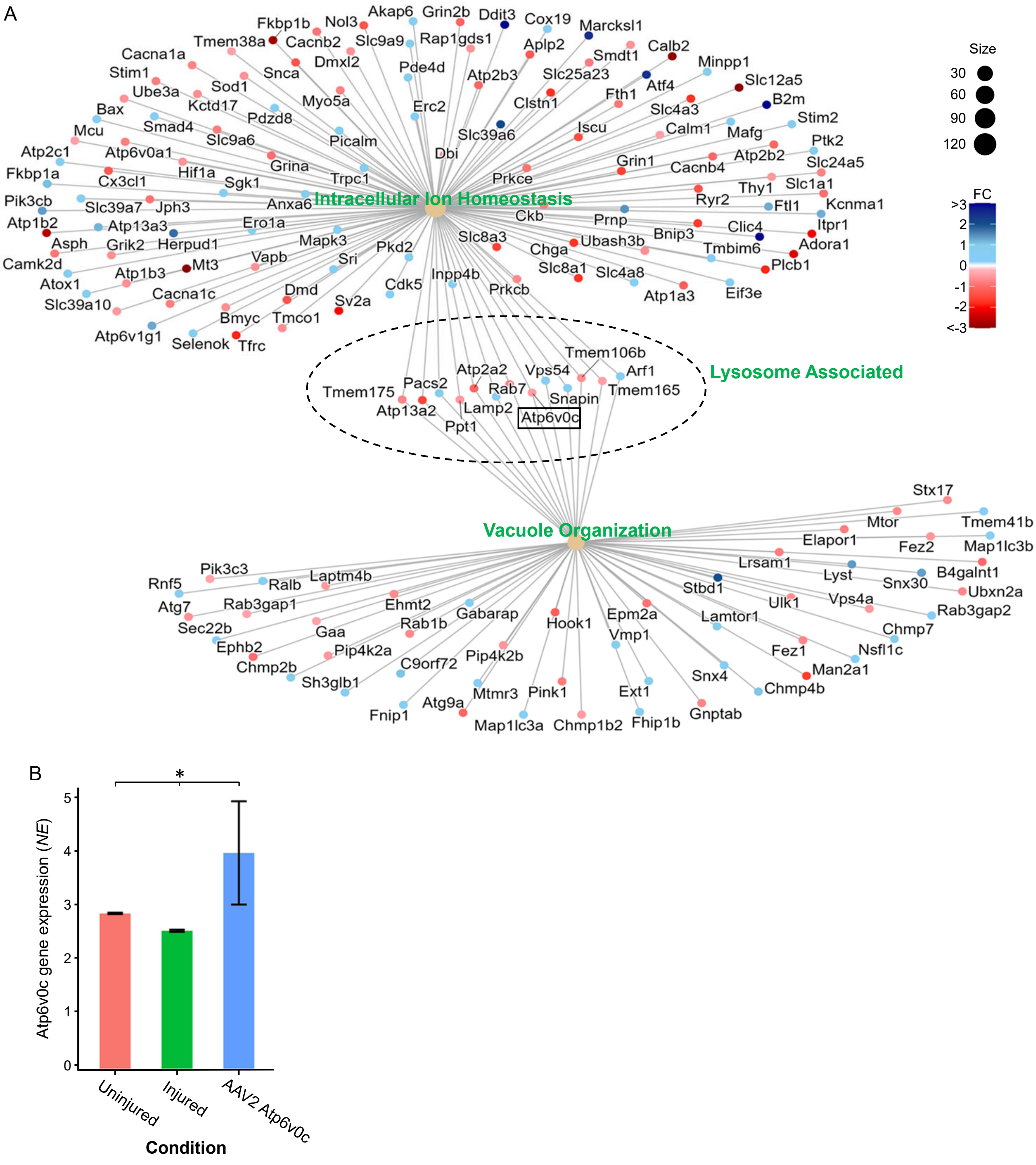

Figure S1. (**A**) Gene-Concept Network Plot of injury-regulated genes associated with GO:BP terms containing Atp6v0c (outlined with a solid line rectangular). GO terms annotated in green font represented by orange circles (*i.e.*, nodes), with circle size (per scale on the side) indicating the number of genes within that node. Color-coded scale bar indicates the log2 fold-change (FC) in expression between adult uninjured and injured RGCs. GO terms' Fold Enrichment was 1.45 (p < 0.001) for Intracellular Ion Homeostasis and 1.66 (p < 0.001) for Vacuole Organization. Shared genes between these two GO terms, outlined by a dashed oval, are manually curated as Lysosome Associated based on literature review. (**B**) Average gene expression of Atp6v0c is significantly increased in AAV2-Atp6v0c-treated injured RGCs relative to both uninjured and injured-control RGCs. scRNA-seq reads mapping to the AAV2 vector transgene UTRs were detected only in the AAV2-Atp6v0c-treated condition, in which endogenous and transgene Atp6v0c were combined for determining *NE* value. Mean (SEM) of *NE* values (log-normalized) are shown; significant differences (p < 0.05, indicated by an asterisk) by ANOVA with posthoc LSD.

SUPPLEMENTAL REFERENCES

1. Hass DT, Barnstable CJ. Mitochondrial Uncoupling Protein 2 Knock-out Promotes Mitophagy to Decrease Retinal Ganglion Cell Death in a Mouse Model of Glaucoma. *J Neurosci*. May 01 2019;39(18):3582-3596. doi:10.1523/JNEUROSCI.2702-18.2019

2. de Lima S, Koriyama Y, Kurimoto T, Oliveira JT, Yin Y, Li Y, Gilbert H, Fagiolini N, Martinez AMB, and Benowitz L. Full-length axon regeneration in the adult mouse optic nerve and partial recovery of simple visual behaviors. *Proc Natl Acad Sci U S A*. Jun 2012;109(23):9149-54. doi:10.1073/pnas.1119449109

3. Rheaume BA, Xing J, Lukomska A, Theune WC, Damania A, Sjogren G, and Trakhtenberg EF. Pten inhibition dedifferentiates long-distance axon-regenerating intrinsically photosensitive retinal ganglion cells and upregulates mitochondria-associated Dynlt1a and Lars2. *Development*. Apr 15 2023;150(8)doi:10.1242/dev.201644

4. Xing J, Theune WC, Lukomska A, Frost MP, Damania A, and Trakhtenberg EF. Experimental upregulation of developmentally downregulated ribosomal protein large subunits 7 and 7A promotes axon regeneration after injury in vivo. *Exp Neurol*. Aug 24 2023:114510. doi:10.1016/j.expneurol.2023.114510

5. Lukomska A, Frost MP, Theune WC, Xing J, Gupta M, and Trakhtenberg EF. Nfe2l3 promotes neuroprotection and long-distance axon regeneration after injury in vivo. *Exp Neurol*. Feb 21 2024:114741. doi:10.1016/j.expneurol.2024.114741

6. Stuart T, Butler A, Hoffman P, Hafemeister C, Papalexi E, Mauck WM 3rd, Hao Y, Stoeckius N, Smibert P, and Satija R. Comprehensive Integration of Single-Cell Data. *Cell*. 06 2019;177(7):1888-1902.e21. doi:10.1016/j.cell.2019.05.031

7. Hao Y, Hao S, Andersen-Nissen E, Mauck WM 3rd, Zheng S, Butler A, Lee MJ, Wilk AJ, Darby C, Zager M, et al. Integrated analysis of multimodal single-cell data. *Cell*. 06 24 2021;184(13):3573-3587.e29. doi:10.1016/j.cell.2021.04.048

8. Mi H, Muruganujan A, Casagrande JT, Thomas PD. Large-scale gene function analysis with the PANTHER classification system. *Nat Protoc*. Aug 2013;8(8):1551-66. doi:10.1038/nprot.2013.092

9. Wu T, Hu E, Xu S, Chen M, Guo P, Dai Z, Feng T, Zhou L, Tang W, Zhan L, et al. clusterProfiler 4.0: A universal enrichment tool for interpreting omics data. *Innovation (Camb)*. Aug 28 2021;2(3):100141. doi:10.1016/j.xinn.2021.100141

10. Theune WC, Frost MP, Trakhtenberg EF. Transcriptomic profiling of retinal cells reveals a subpopulation of microglia/macrophages expressing Rbpms marker of retinal ganglion cells (RGCs) that confound identification of RGCs. *Brain Res*. Jul 15 2023;1811:148377. doi:10.1016/j.brainres.2023.148377

11. Trakhtenberg EF. Single-cell transcriptomics-enabled advances in experimental optic nerve axon regeneration research. In: Bhattacharya SK, ed. *Proteomics, Multi-Omics and Systems Biology in Optic Nerve Regeneration*. Academic Press; 2025:207-223:chap 14.

12. Rheaume BA, Jereen A, Bolisetty M, Sajid MS, Yang Y, Renna K, Sun L, Robson P, and Trakhtenberg EF. Single cell transcriptome profiling of retinal ganglion cells identifies cellular subtypes. *Nat Commun*. Jul 2018;9(1):2759. doi:10.1038/s41467-018-05134-3
