# Supplementary material for: Vacuolar ATPase subunit Atp6v0c transgene promotes neuroprotection and long-distance axon regeneration in injured retinal ganglion neurons
Source: Mol Ther Nucleic Acids. 2026 Apr 1;37(2):102922. doi: 10.1016/j.omtn.2026.102922 (PMC13096995; doi:10.1016/j.omtn.2026.102922)
Supplement: Document S2. Article plus supplemental information [file mmc2.pdf]

Molecular Therapy
Nucleic Acids
Brief report

Vacuolar ATPase subunit Atp6v0c transgene promotes neuroprotection and long-distance axon regeneration in injured retinal ganglion neurons

Anja Kearney,[1,2] Agnieszka Lukomska,[1,2] Jacob Brady,[1] Ashiti Damania,[1] Mahit Gupta,[1] and Ephraim F. Trakhtenberg[1]

[1]Department of Neuroscience, University of Connecticut School of Medicine, 263 Farmington Ave, Farmington, CT 06030, USA

Central nervous system (CNS) projection neurons' failure to repair or regenerate injured axons has devastating consequences for those who have sustained CNS injuries. Thus, there is a need for translatable factors capable of promoting long-distance axon regeneration in the CNS. We hypothesized that supporting lysosomes in injured neurons by supplementing their structural factors through gene therapy may foster axon regeneration. To test our hypothesis, we selected Atp6v0c for experimental regulation because it plays roles in lysosomal acidification and the degradation of misfolded proteins in response to endoplasmic reticulum (ER) stress in injured neurons. We tested this in a rodent optic nerve crush (ONC) model of traumatic optic neuropathy (TON), in which injured prototypical CNS projection neurons, the retinal ganglion cells (RGCs), do not regenerate damaged axons and eventually degenerate. Atp6v0c transgene expression was achieved using intravitreally injected adeno-associated virus serotype 2 (AAV2), which transduces the RGCs. For benchmarking, we compared efficacy to AAV2 targeting of prominent regulators of axon regeneration, Pten, and Klf9. We found that Atp6v0c transgene promoted RGC survival and long-distance axon regeneration, comparable to targeting Pten and Klf9. Thus, Atp6v0c is an axon regeneration-promoting factor with potential for treating CNS injury and disease.

INTRODUCTION

Central nervous system (CNS) projection neurons' failure to repair and regenerate injured axons has devastating consequences for those who have sustained spinal cord injury, stroke, brain trauma, or optic neuropathy. Axonal self-repair and regeneration failure in the CNS affects mammals, but not necessarily lower vertebrates, and therefore rodent CNS injury models have been developed to tackle this problem. Like other CNS projection neurons, the retinal ganglion cells (RGCs) do not spontaneously regenerate injured optic nerve axons in an established rodent optic nerve crush (ONC) model of traumatic optic neuropathy (TON).[1] Oxidative/ endoplasmic reticulum (ER) stress, implicated in neuronal response to injury, is among the earliest pathological events in RGCs after ONC.[2] Several antioxidant factors are neuroprotective for neuronal survival, but with a few exceptions, they do not generally promote substantial optic nerve axon regeneration *in vivo*. ER stress activates the unfolded protein response, which can be neuroprotective in traumatic/ischemic brain injuries and neurodegenerative conditions.[3] ER stress also activates lysosomal degradation of misfolded proteins,[4] and targeting ER-stress-induced lysosomal overload is neuroprotective.[5] Furthermore, reducing transport of degradative lysosomes into axons leads to degeneration,[6–8] while augmenting lysosomal degradation fosters axonal self-repair.[9,10] Thus, we hypothesized that supporting lysosomes in injured neurons by supplementing their structural factors through RGC-targeted gene therapy may foster long-distance axon regeneration after ONC *in vivo*. This would imply that a structural factor, established to be critical for lysosomal function, which is dysregulated by axonal injury, might be a viable neurotherapeutic target.

In order to test this implication, we selected Atp6v0c for experimental targeting because (a) it plays roles in lysosomal acidification and degradation of misfolded proteins in response to ER stress[11] and (b) we found it linked to dysregulation of the lysosome-associated gene network in the injured RGCs (Figure S1A). Atp6v0c is a critical subunit of the vacuolar ATPase (V-ATPase) complex, which is an ATP-driven proton pump that acidifies various vacuolar/vesicular intracellular organelles[12,13] and mediates autophagosome/lysosome-mediated degradation of pathogenic proteins in neurodegenerative diseases.[14] V-ATPase consists of two multi-subunit domains, with the V1 ATPase domain powering the Vo proton-pump domain. The Vo complex itself is comprised of the c-ring core, through which protons are transferred for acidification of the lumen, and supporting subunits (*a*, *d*, *e*, RNAseK, Atp6ap1, Atp6ap2), which interact with the c-core. The c-ring subunits are encoded by two genes, with Atp6v0b contributing 1 *c*" subunit and Atp6v0c contributing 9 identical *c* subunits, with all 10 vertically aligning next to each other in a closed circle, forming a c-ring[15] (see below). Association of

Received 21 September 2025; accepted 27 March 2026;
https://doi.org/10.1016/j.omtn.2026.102922.

[2]These authors contributed equally

Correspondence: Ephraim F. Trakhtenberg, Department of Neuroscience, University of Connecticut School of Medicine, 263 Farmington Ave, Farmington, CT 06030, USA.
E-mail: trakhtenberg@uchc.edu

© 2026 The Author(s).
Published by Elsevier Inc. on behalf of The American Society of Gene and Cell Therapy.
This is an open access article under the CC BY license (http://creativecommons.org/licenses/by/4.0/).

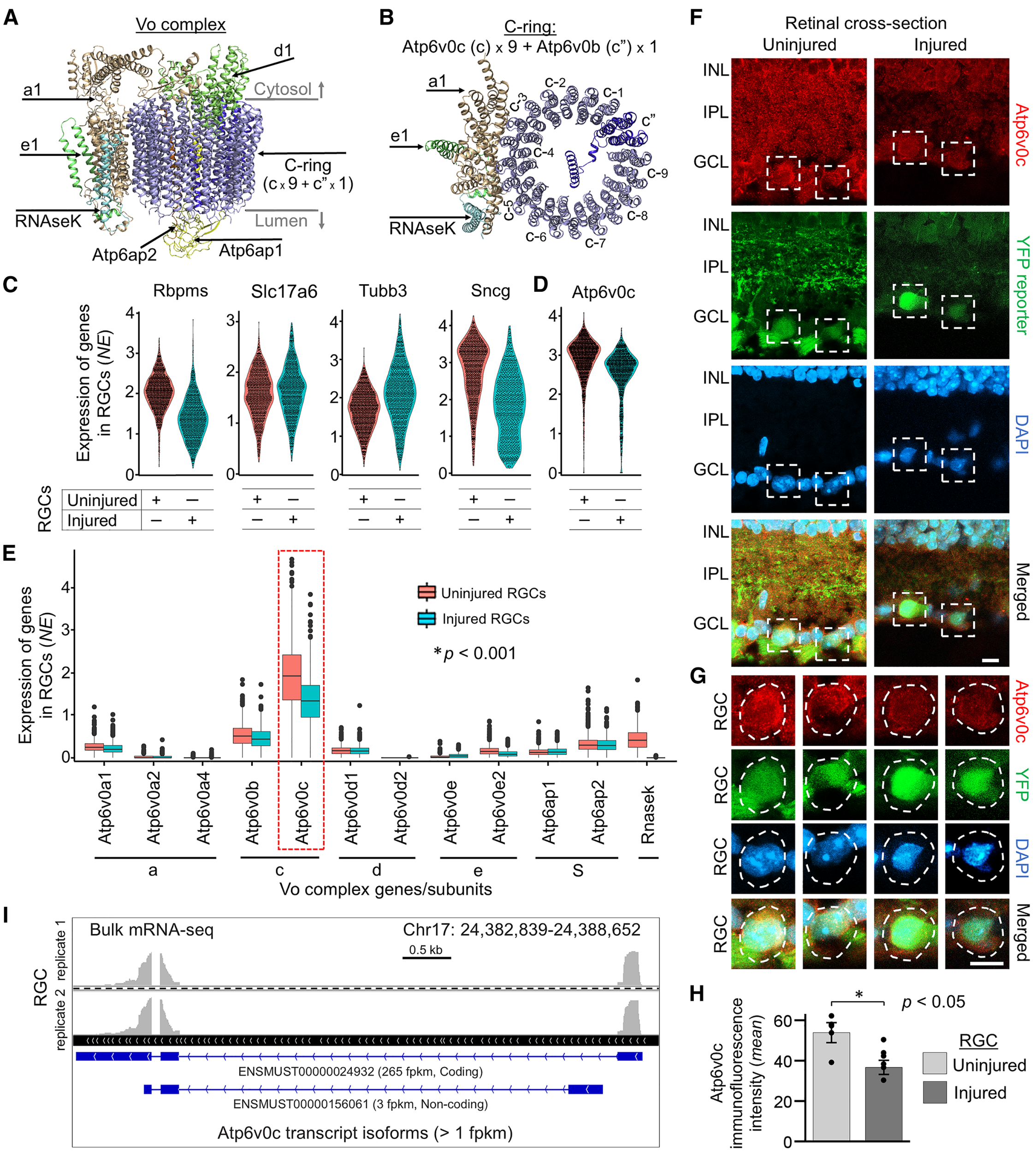

Figure 1. Expression of the V-ATPase Atp6v0c subunit gene is downregulated in RGCs after optic nerve injury

(A and B) Schematic of the V-ATPase Vo transmembrane domain, lateral (*A*) and horizontal (*B*) views shown; redrawn from Wang et al. (2020).[15] Vo complex subunits are indicated, as marked. The c-ring, through which proton is transferred, consists of 9× *c* subunits, encoded by the Atp6v0c gene, and 1× *c*" subunit, encoded by the Atp6v0c gene, as marked (*B*). (C) Gene expression violin plots of pan-RGC marker genes in scRNA-seq-profiled cells, as marked, show that these markers are robustly expressed in both uninjured and injured conditions, confirming RGC cell-type identity. (D) Gene expression violin plot of Atp6v0c in scRNA-seq-profiled RGCs shows that Atp6v0c is downregulated in injured compared to uninjured RGCs. (E) Boxplots showing V-ATPase Vo complex subunit genes expression in scRNA-seq-profiled RGCs before and 2 weeks after ONC injury, as marked. Median ± interquartile range error bars of normalized expression (*NE*) values (not logged) are shown. Statistical significance of differential gene expression was determined by ANOVA (overall $F = 5317$, $p < 0.001$, with p values of pairwise comparisons determined by post hoc least significant difference [LSD]). Pairwise differences were significant between uninjured and injured conditions for Atp6v0c ($p < 0.001$), Rnasek ($p < 0.001$), Atp6v0e ($p < 0.01$), and some other genes (not specified due to a small fold change and overall low expression levels); n represents individual scRNA-seq-profiled RGCs ($n = 1683$ in uninjured and $n = 1375$ in the injured condition; see materials and methods). (F–H) Confocal microscopy with a 63× objective was used to determine co-localization of Atp6v0c immunofluorescence signal and RGC reporter yellow fluorescent protein (YFP) in the same cells. Representative images of the immunostained retinal cross-sections (*F*), along with higher magnification insets (RGCs outlined with dashed lines; *G*), show reductions in both Atp6v0c granules and diffuse immunofluorescence signal in ONC-injured, compared to uninured, RGCs (in the Thy1-YFP mouse strain, YFP labels the RGCs within the retinal ganglion cell layer; GCL[23]). Apparent reduction of Atp6v0c signal in the inner plexiform layer (IPL) is consistent

(legend continued on next page)

various disorders with deficiencies in Atp6v0c is consistent with this subunit's major contribution to the critical c-ring. For example, Atp6v0c loss-of-function mutations are embryonically lethal,[16] while abnormalities in Atp6v0c function are associated with neurodegenerative diseases and lead to dysregulation of autophagy and the lysosomal degradation pathway,[17] including in Parkinson's[18] and Alzheimer's[19,20] diseases. Atp6v0c is also involved in the maintenance of synaptic vesicles,[21] and its dysregulation results in seizures.[22] However, it is unknown whether experimental upregulation of Atp6v0c in injured CNS neurons could promote regeneration of damaged axons.

RESULTS

First, we utilized single cell RNA-seq (scRNA-seq) to characterize whether expression of any of the V-ATPase's Vo complex subunit members (shown in Figures 1A and 1B) is altered in RGCs after ONC injury. Based on expression of the specific Vo complex genes in scRNA-seq-profiled adult uninjured and 2 weeks post-ONC injured RGCs (cell-type identity confirmed using established pan-RGC markers[24]; Figure 1C), we found that a risk-predisposing change occurred in Atp6v0c expression, as it was significantly downregulated in RGCs after injury (Figures 1D and 1E). While Atp6v0e, encoding subunit *e*, was upregulated after injury, an increase in expression does not pose an apparent loss-of-function risk and may also compensate for downregulation of an alternative *e* subunit isoform, Atp6v0e2, which itself is not high risk, as it is neither ubiquitous nor associated with neurological disorders.[25] The downregulated RNAseK subunit, however, is another potential target, which we did not pursue here, as it has not been previously associated with any neurological disorders[26] (Figure 1E). We validated Atp6v0c protein downregulation in the injured RGCs by immunostaining, which showed a significant post-injury decrease (Figures 1F–1H). Considering that Atp6v0c contributes 9 out of 10 c-ring subunits, its downregulation and dysregulation in injured RGCs may hinder self-recovery/repair ability following injury-induced cellular stress, which is consistent with the association between Atp6v0c deficiencies and neurological disorders.[18–20,22] We then used bulk-mRNA-seq-profiling of adult RGCs to identify which Atp6v0c protein-coding transcript isoforms are expressed in RGCs, in order to select an open reading frame (ORF) for experimental transgene vector design (Figure 1I).

Next, we tested whether sustained upregulation of Atp6v0c in injured RGCs is sufficient to promote axon regeneration after ONC. For experimental Atp6v0c upregulation, we selected from the transcript-specific bulk-mRNA-seq-profiled RGC dataset an ORF of the only detected protein-coding Atp6v0c transcript identified in both replicates (Figure 1I). Stable experimental neuronal Atp6v0c transgene expression was achieved using intravitreally injected adeno-associated virus serotype 2 (AAV2), which transduces the RGCs. For benchmarking, we compared the effects to AAV2 targeting of prominent regulators of long-distance axon regeneration, Pten, and Klf9.[24,27,28] To validate AAV2 vector expression (which transduces RGCs within the retina) in injured RGCs, a Myc reporter was added to the *N*-terminus of the Atp6v0c ORF. For negative control, an mCherry-expressing AAV2 vector was used. For positive control, established axon regeneration-promoting AAV2 vectors expressing short hairpin RNAs (shRNAs) to knock down (KD) expression of Pten or Klf9, and co-expressing an mCherry reporter, were used.[24,28] The viruses were injected intravitreally in adult mice, and 2 weeks later, ONC injury was performed (Figure 2A). Co-immunostaining of the retinas 2 weeks after ONC for the Myc-tag reporter and βIII-Tubulin (a neuronal-specific marker that selectively labels the RGCs within the retina) confirmed expression of the Atp6v0c transgene in the transduced (Myc reporter-labeled) injured RGCs, with transduction efficiency of ∼30%, similar to prior reports that also used AAV2 to target the RGCs[24] (as shown by confocal images in Figures 2B–2E, wider field-of-view images in Figures 2F and 2G, and quantifications in Figure 2H); transgene expression was also validated by scRNA-seq (Figure S1B). mCherry reporter-labeled injured RGCs also confirmed comparable transduction and expression of the established AAV2 vectors used here for negative (mCherry) and positive (anti-Pten and anti-Klf9 shRNAs) controls (Figures 2B–2D and 2H).

Then, we tested whether directly upregulating Atp6v0c in injured RGCs would promote RGC survival and axon regeneration after injury. We used an established 2-week post-ONC axon regeneration assay,[24,27,28] in which the optic nerve is injured by crush in order to sever all RGC axons, and RGC survival and axon regeneration are assayed at 2 weeks after injury. AAV2 vectors expressing Atp6v0c, anti-Pten shRNA, anti-Klf9 shRNA, or mCherry control were injected intravitreally in adult mice, and 2 weeks later, ONC was performed. To visualize the regenerating axons or their absence, the axonal tracer Cholera toxin subunit B (CTB)-488 was injected 1 day prior to sacrifice at 2 weeks after ONC. The number of regenerating axons was quantified in longitudinal sections of the optic nerve (no spared axons were detected in either group), and RGC survival was quantified in retinal flat mounts (see materials and methods for details; experimental timeline in Figure 2A). We found that Atp6v0c upregulation promoted RGC survival by approximately 25%, which is a similar extent of neuroprotection as with anti-Pten shRNA and anti-Klf9 shRNA AAV2 treatments by 2 weeks post-ONC, compared to RGC survival in the injured control group (Figures 2I–2K).

with degeneration or shrinkage of dead or surviving RGC dendrites and consequent thinning of the IPL between the GCL and the inner nuclear layer (INL) by 2 weeks post-ONC. Scale bars, 10 μm (*F-G*). Atp6v0c immunofluorescence signal intensity was quantified in RGCs (YFP+ labeled cells in the GCL) using ZEN software (Zeiss) measurements tools for average pixel intensity (arbitrary units) and analyzed by independent samples *t* test, 2-tailed; significant difference ($p < 0.05$) indicated by an asterisk *. Mean ± SEM shown; n = 5–6 cases per group (*H*). (I) Adult uninjured RGC bulk mRNA-seq normalized read alignment to the Atp6v0c gene is shown along with the detected Ensembl transcript isoforms assembled by Cufflinks and normalized by CuffDiff expression values (fpkm), with the protein-coding and non-coding splice variants specified, as marked (see materials and methods). Visualization with IGV viewer.

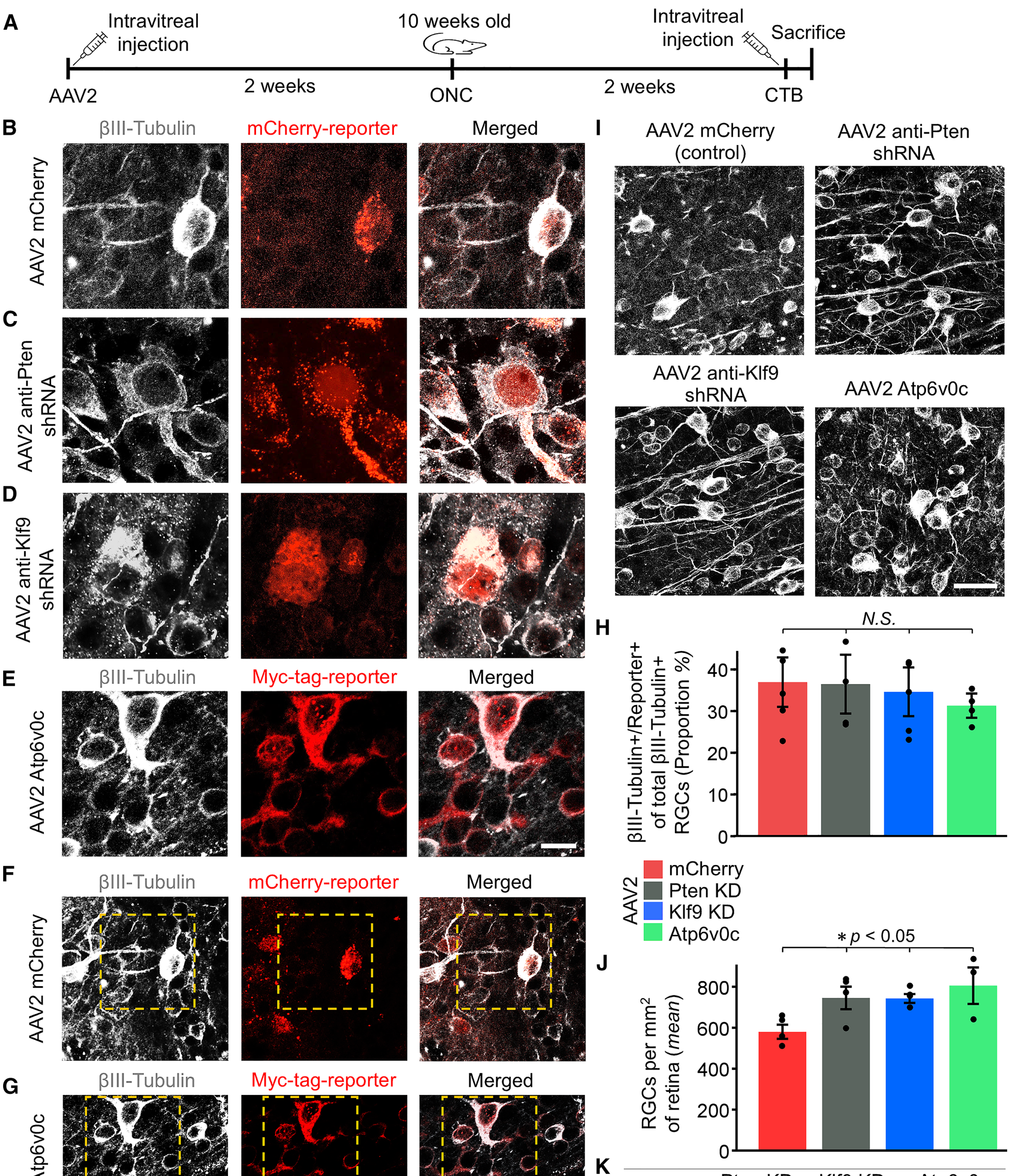

	Pten KD	Klf9 KD	Atp6v0c
mCherry	0.035 *	0.037 *	0.011 *
Pten KD		0.970	0.436
Klf9 KD			0.417

Figure 2. Atp6v0c transgene promotes RGC survival after optic nerve injury

(A) Experimental timeline: AAV2 viral vectors were injected intravitreally in 8-week-old mice, and 2 weeks later, ONC injury was performed. Animals were sacrificed for histological analysis 2 weeks after ONC. For axon regeneration studies (shown in Figure 3), the axonal tracer CTB was injected intravitreally 1 day prior to sacrifice. (B–E) Confocal microscopy with a 63× objective was used to determine co-localization of vector reporters and RGC marker in the same cells. High-magnification representative images of the immunostained retinal flat mounts' GCL show that mCherry and Myc-tag reporters of transduction/expression localize to βIII-Tubulin+ cells (βIII-Tubulin is a neuronal-specific marker that labels the RGCs within the retina), and that transduced cells are detected at similar proportions in all conditions, as marked (*B-E*). Scale bars, 10 μm. (F and G) Lower-magnification, larger field-of-view representative images for the control with mCherry reporter (*F*; dashed-line outlined inset shown in *B*) and for the Atp6v0c transgene with mCherry Myc-tag reporter (*G*; dashed-line outlined inset shown in *E*) vectors, processed as above. Scale bars, 10 μm. (H) Proportion of βIII-Tubulin+/

(legend continued on next page)

Although, given that over 80% of RGCs die by 2 weeks after ONC,[29] the observed increase in survival is modest (as only about 5% fewer RGCs died), if experimental neuroprotection persists permanently, this would be meaningful because, without treatment, most or nearly all RGCs eventually die. Importantly, relative to only minor axonal sprouting past the injury site detected in control animals (as expected), the Atp6v0c transgene promoted long-distance axon regeneration past the injury site, which is comparable to the extent of axon regeneration achieved by anti-Pten shRNA and anti-Klf9 shRNA AAV2 treatments (Figures 3A–3G).

DISCUSSION

No clinical treatments exist for regenerating damaged axons, limiting recovery options for CNS injuries. Toward addressing this unmet medical need, using gene therapy for targeting a transgene to injured neurons, we identified the V-ATPase subunit Atp6v0c as an axon regeneration-promoting factor with neurotherapeutic potential for CNS injury and disease. Remarkably, the extent of neuroprotection and axon regeneration was comparable to the effects of AAV2 targeting Pten and Klf9, which are benchmark regulators of CNS axon regeneration.[24,27,28] Although conditional genetic knockout of Pten leads to greater survival and axon regeneration than AAV2-mediated KD,[27] we used the same viral vector delivery method in all conditions for a more appropriate comparison of the treatments *per se*, which better reflects their translational potential via gene therapy.

The limitations and future directions are as follows. For a proof of concept, we used the standard in the field 2-week post-ONC time point to assess whether the Atp6v0c transgene has neurotherapeutic potential. Future long-term studies are needed to explore the full potential, as prior studies suggested that ∼3 months are needed post-ONC for experimentality stimulated regenerating axons to reach post-synaptic targets in respective brain nuclei for recovery of visual functions.[30,31] Future long-term studies should also examine Atp6v0c levels at additional time points post-injury and explore whether treatment effects vary across RGC subtypes or retinal regions. Additionally, leveraging emerging improved AAV2-based vectors with higher transduction efficiency could further enhance therapeutic efficacy. Furthermore, as a greater extent of axon regeneration is achievable by combining different approaches,[30,31] future studies may find that Atp6v0c upregulation cooperates with other treatments to yield an even greater extent of axonal repair/regeneration. A number of effectors may be implicated in the neurotherapeutic efficacy we observed. For example, enhancing lysosomal function in injured neurons may promote axon regeneration by facilitating a higher demand for lysosomal degradation of proteins that misfolded either due to ER stress or an increased protein synthesis demand for supporting axonal regeneration in a pathological microenvironment. This could be particularly relevant for the degradative lysosomes transported for function into damaged axons. Exploring these possibilities, along with the upstream and downstream molecular mechanisms, represents an important future research direction. For example, future studies could explore whether the Atp6v0c transgene rescues a specific lysosomal dysfunction by mitigating ER stress, regulating autophagy, and/or improving lysosomal acidification and clearing misfolded proteins. Moreover, because previous association of Atp6v0c with epilepsy and neurodevelopmental disorders was due to congenital mutations in the Atp6v0c gene,[22] upregulating wild-type Atp6v0c is not apparently concerning; nevertheless, future translational studies would need to evaluate the safety of increasing Atp6v0c levels in injured neurons.

MATERIALS AND METHODS

Materials And Methods are provided in the supplemental information.

DATA AND CODE AVAILABILITY

- The plasmid for the Atp6v0c AAV2 vector will be shared for academic purposes. This study did not generate any other new unique reagents.
- RNA sequencing data from this study have been deposited in the Gene Expression Omnibus (GEO) at the NCBI repository and are publicly available (accession number GSE325128).
- This paper does not report original code.
- Any additional information required to reanalyze the data reported in this paper is available from the lead contact upon request.

ACKNOWLEDGMENTS

This work was supported by a grant from the National Institutes of Health (NIH), National Eye Institute (NEI) (grant R01-EY029739, to E.F.T.). Portions of this research were conducted at the High Performance Computing Facility, University of Connecticut. We are grateful to Bill Flynn and Elise Courtois (The Jackson Laboratory for Genomic Medicine, Farmington, CT) for single-cell RNA-sequencing service and to Sophan Iv (Research IT Services, University of Connecticut) and Stephen King (High Performance Computing Facility, University of Connecticut) for bioinformatics support. We thank Mathew Frost, Madison Sakheim, and Lucy Homer (students, University of Connecticut School of Medicine) for technical assistance.

AUTHOR CONTRIBUTIONS

A.K.[#], A.L.[#], J.B., A.D., and M.G., performed the experiments. E.F.T. designed the study and wrote the manuscript.

DECLARATION OF INTERESTS

The authors declare no competing interests.

SUPPLEMENTAL INFORMATION

Supplemental information can be found online at https://doi.org/10.1016/j.omtn.2026.102922.

Reporter+ cells among total βIII-Tubulin+ RGCs (%) in the GCL, analyzed by ANOVA with post hoc LSD. No significant differences were found. Mean ± SEM shown; $n = 4$ cases per group. *N.S.* = not significant. (I) Representative images of retinal flat mounts' GCL immunostained for an RGC marker βIII-Tubulin at 2 weeks after ONC, pre-treated with experimental and control AAV2 vectors, as marked. Sale bars, 20 μm. (J and K) Quantitation of RGC (βIII-Tubulin+ cells) survival in retinal flat mounts' GCL at 2 weeks after ONC, from the experimental and control treatment conditions, as marked. Mean ± SEM) shown; $n = 3–4$ cases per group (*J*). Data analyzed using ANOVA, overall $F = 3.68$, $p < 0.05$, with p values of pairwise comparisons determined by post hoc LSD; significant differences ($p < 0.05$) indicated by an asterisk * (*K*).

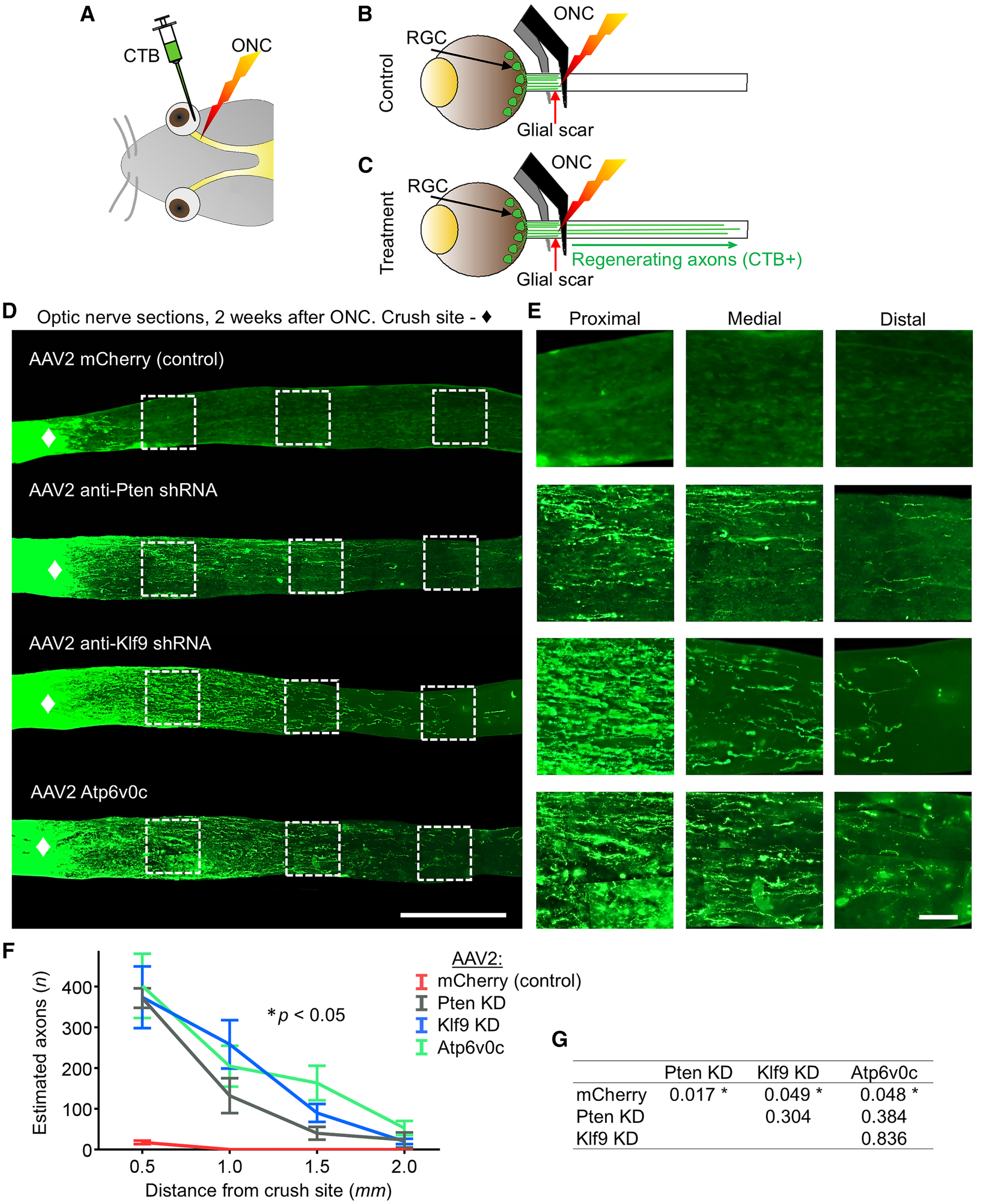

	Pten KD	Klf9 KD	Atp6v0c
mCherry	0.017 *	0.049 *	0.048 *
Pten KD		0.304	0.384
Klf9 KD			0.836

Figure 3. Atp6v0c transgene promotes axon regeneration after optic nerve injury

(A–C) Experimental timeline shown in Figure 2A. For visualization of the regenerating axons, or their absence, an axonal tracer CTB (conjugated to Alexa Fluor 488 dye) is injected intravitreally 1 day before sacrifice. It is rapidly taken by the RGCs and transported anterogradely along the intact axons that are connected to RGC soma (*A*). In the control condition, the CTB-labeled axons end abruptly at the ONC injury site (*B*), but in the experimental treatment condition, the regenerating axons are CTB-labeled also beyond the injury site, to the length they regrew along the optic nerve in 2 weeks (a standard endpoint in axon regeneration proof-of-concept studies) (*C*). (D and E) Representative images of optic nerve longitudinal cryosections with CTB-labeled axons at 2 weeks after ONC from animals pre-treated with experimental and control AAV2 vectors, as marked (*D*). Insets: Representative images of the optic nerve regions proximal and distal to the injury site are magnified for better visualization of the axons or their absence (*E*). The edges of the tissue were optically trimmed (i.e., cropped) due to artifactual autofluorescence that is common at tissue edges (see materials and methods). Scale bars, 500 μm (main panels), 50 μm (insets). (F and G) Quantitation of CTB-labeled regenerating axons at 2 weeks after ONC, at increasing distances from the injury site, after pre-treatment with experimental and control AAV2 vectors, as marked. Mean ± SEM shown; n = 3–4 cases per group (*F*). Data analyzed using repeated-measures ANOVA with post hoc LSD, sphericity assumed, overall $F = 6.28$, $p < 0.05$; post hoc p value ($p < 0.05$) indicated by an asterisk * (*G*).

REFERENCES

1. Ghaffarieh, A., and Levin, L.A. (2012). Optic nerve disease and axon pathophysiology. Int. Rev. Neurobiol. *105*, 1–17. https://doi.org/10.1016/B978-0-12-398309-1.00002-0.
2. Kanamori, A., Catrinescu, M.M., Kanamori, N., Mears, K.A., Beaubien, R., and Levin, L.A. (2010). Superoxide is an associated signal for apoptosis in axonal injury. Brain *133*, 2612–2625. https://doi.org/10.1093/brain/awq105.
3. Hetz, C., and Saxena, S. (2017). ER stress and the unfolded protein response in neurodegeneration. Nat. Rev. Neurol. *13*, 477–491. https://doi.org/10.1038/nrneurol.2017.99.
4. Reggiori, F., and Molinari, M. (2022). ER-phagy: mechanisms, regulation, and diseases connected to the lysosomal clearance of the endoplasmic reticulum. Physiol. Rev. *102*, 1393–1448. https://doi.org/10.1152/physrev.00038.2021.
5. Bisicchia, E., Mastrantonio, R., Nobili, A., Palazzo, C., La Barbera, L., Latini, L., Millozzi, F., Sasso, V., Palacios, D., D'Amelio, M., and Viscomi, M.T. (2022). Restoration of ER proteostasis attenuates remote apoptotic cell death after spinal cord injury by reducing autophagosome overload. Cell Death Dis. *13*, 381. https://doi.org/10.1038/s41419-022-04830-9.
6. Farfel-Becker, T., Roney, J.C., Cheng, X.T., Li, S., Cuddy, S.R., and Sheng, Z.H. (2019). Neuronal Soma-Derived Degradative Lysosomes Are Continuously Delivered to Distal Axons to Maintain Local Degradation Capacity. Cell Rep. *28*, 51–64.e4. https://doi.org/10.1016/j.celrep.2019.06.013.
7. Roney, J.C., Cheng, X.T., and Sheng, Z.H. (2022). Neuronal endolysosomal transport and lysosomal functionality in maintaining axonostasis. J. Cell Biol. *221*, e202111077. https://doi.org/10.1083/jcb.202111077.
8. Maday, S. (2016). Mechanisms of neuronal homeostasis: Autophagy in the axon. Brain Res. *1649*, 143–150. https://doi.org/10.1016/j.brainres.2016.03.047.
9. He, M., Ding, Y., Chu, C., Tang, J., Xiao, Q., and Luo, Z.G. (2016). Autophagy induction stabilizes microtubules and promotes axon regeneration after spinal cord injury. Proc. Natl. Acad. Sci. USA *113*, 11324–11329. https://doi.org/10.1073/pnas.1611282113.
10. Ko, S.H., Apple, E.C., Liu, Z., and Chen, L. (2020). Age-dependent autophagy induction after injury promotes axon regeneration by limiting NOTCH. Autophagy *16*, 2052–2068. https://doi.org/10.1080/15548627.2020.1713645.
11. Sun, Y., Wang, X., Yang, X., Wang, L., Ding, J., Wang, C.C., Zhang, H., and Wang, X. (2023). V-ATPase recruitment to ER exit sites switches COPII-mediated transport to lysosomal degradation. Dev. Cell *58*, 2761–2775.e5. https://doi.org/10.1016/j.devcel.2023.10.007.
12. Forgac, M. (2007). Vacuolar ATPases: rotary proton pumps in physiology and pathophysiology. Nat. Rev. Mol. Cell Biol. *8*, 917–929. https://doi.org/10.1038/nrm2272.
13. Zhao, J., Benlekbir, S., and Rubinstein, J.L. (2015). Electron cryomicroscopy observation of rotational states in a eukaryotic V-ATPase. Nature *521*, 241–245. https://doi.org/10.1038/nature14365.
14. Lee, J.H., Yu, W.H., Kumar, A., Lee, S., Mohan, P.S., Peterhoff, C.M., Wolfe, D.M., Martinez-Vicente, M., Massey, A.C., Sovak, G., et al. (2010). Lysosomal proteolysis and autophagy require presenilin 1 and are disrupted by Alzheimer-related PS1 mutations. Cell *141*, 1146–1158. https://doi.org/10.1016/j.cell.2010.05.008.
15. Wang, L., Wu, D., Robinson, C.V., Wu, H., and Fu, T.M. (2020). Structures of a Complete Human V-ATPase Reveal Mechanisms of Its Assembly. Mol. Cell *80*, 501–511.e3. https://doi.org/10.1016/j.molcel.2020.09.029.
16. Aoto, K., Kato, M., Akita, T., Nakashima, M., Mutoh, H., Akasaka, N., Tohyama, J., Nomura, Y., Hoshino, K., Ago, Y., et al. (2021). ATP6V0A1 encoding the a1-subunit of the V0 domain of vacuolar H. Nat. Commun. *12*, 2107. https://doi.org/10.1038/s41467-021-22389-5.
17. Mangieri, L.R., Mader, B.J., Thomas, C.E., Taylor, C.A., Luker, A.M., Tse, T.E., Huisingh, C., and Shacka, J.J. (2014). ATP6V0C knockdown in neuroblastoma cells alters autophagy-lysosome pathway function and metabolism of proteins that accumulate in neurodegenerative disease. PLoS One *9*, e93257. https://doi.org/10.1371/journal.pone.0093257.
18. George, J., Shafiq, K., Kapadia, M., Kalia, L.V., and Kalia, S.K. (2024). High frequency electrical stimulation reduces α-synuclein levels and α-synuclein-mediated autophagy dysfunction. Sci. Rep. *14*, 16091. https://doi.org/10.1038/s41598-024-64131-3.
19. Liu, Q.Y., Lei, J.X., Sikorska, M., and Liu, R. (2008). A novel brain-enriched E3 ubiquitin ligase RNF182 is up regulated in the brains of Alzheimer's patients and targets ATP6V0C for degradation. Mol. Neurodegener. *3*, 4. https://doi.org/10.1186/1750-1326-3-4.
20. Kim, S.H., Cho, Y.S., Kim, Y., Park, J., Yoo, S.M., Gwak, J., Kim, Y., Gwon, Y., Kam, T.I., and Jung, Y.K. (2023). Endolysosomal impairment by binding of amyloid beta or MAPT/Tau to V-ATPase and rescue via the HYAL-CD44 axis in Alzheimer disease. Autophagy *19*, 2318–2337. https://doi.org/10.1080/15548627.2023.2181614.
21. Abbas, Y.M., Wu, D., Bueler, S.A., Robinson, C.V., and Rubinstein, J.L. (2020). Structure of V-ATPase from the mammalian brain. Science *367*, 1240–1246. https://doi.org/10.1126/science.aaz2924.
22. Mattison, K.A., Tossing, G., Mulroe, F., Simmons, C., Butler, K.M., Schreiber, A., Alsadah, A., Neilson, D.E., Naess, K., Wedell, A., et al. (2023). ATP6V0C variants impair V-ATPase function causing a neurodevelopmental disorder often associated with epilepsy. Brain *146*, 1357–1372. https://doi.org/10.1093/brain/awac330.
23. Hass, D.T., and Barnstable, C.J. (2019). Mitochondrial Uncoupling Protein 2 Knock-out Promotes Mitophagy to Decrease Retinal Ganglion Cell Death in a Mouse Model of Glaucoma. J. Neurosci. *39*, 3582–3596. https://doi.org/10.1523/JNEUROSCI.2702-18.2019.
24. Rheaume, B.A., Xing, J., Lukomska, A., Theune, W.C., Damania, A., Sjogren, G., and Trakhtenberg, E.F. (2023). Pten inhibition dedifferentiates long-distance axon-regenerating intrinsically photosensitive retinal ganglion cells and upregulates mitochondria-associated Dynlt1a and Lars2. Development *150*, dev201644. https://doi.org/10.1242/dev.201644.
25. Blake-Palmer, K.G., Su, Y., Smith, A.N., and Karet, F.E. (2007). Molecular cloning and characterization of a novel form of the human vacuolar H+-ATPase e-subunit: an essential proton pump component. Gene *393*, 94–100. https://doi.org/10.1016/j.gene.2007.01.020.
26. Makar, A.N., Boraman, A., Mosen, P., Simpson, J.E., Marques, J., Michelberger, T., Aitken, S., Wheeler, A.P., Winter, D., von Kriegsheim, A., and Gammoh, N. (2024). The V-ATPase complex component RNAseK is required for lysosomal hydrolase delivery and autophagosome degradation. Nat. Commun. *15*, 7743. https://doi.org/10.1038/s41467-024-52049-3.
27. Park, K.K., Liu, K., Hu, Y., Smith, P.D., Wang, C., Cai, B., Xu, B., Connolly, L., Kramvis, I., Sahin, M., and He, Z. (2008). Promoting axon regeneration in the adult CNS by modulation of the PTEN/mTOR pathway. Science *322*, 963–966.
28. Apara, A., Galvao, J., Wang, Y., Blackmore, M., Trillo, A., Iwao, K., Brown, D.P., Fernandes, K.A., Huang, A., Nguyen, T., et al. (2017). KLF9 and JNK3 Interact to Suppress Axon Regeneration in the Adult CNS. J. Neurosci. *37*, 9632–9644. https://doi.org/10.1523/JNEUROSCI.0643-16.2017.
29. Mead, B., and Tomarev, S. (2016). Evaluating retinal ganglion cell loss and dysfunction. Exp. Eye Res. *151*, 96–106. https://doi.org/10.1016/j.exer.2016.08.006.
30. de Lima, S., Koriyama, Y., Kurimoto, T., Oliveira, J.T., Yin, Y., Li, Y., Gilbert, H.Y., Fagiolini, M., Martinez, A.M.B., and Benowitz, L. (2012). Full-length axon regeneration in the adult mouse optic nerve and partial recovery of simple visual behaviors. Proc. Natl. Acad. Sci. USA *109*, 9149–9154. https://doi.org/10.1073/pnas.1119449109.
31. Lim, J.H.A., Stafford, B.K., Nguyen, P.L., Lien, B.V., Wang, C., Zukor, K., He, Z., and Huberman, A.D. (2016). Neural activity promotes long-distance, target-specific regeneration of adult retinal axons. Nat. Neurosci. *19*, 1073–1084. https://doi.org/10.1038/nn.4340.

OMTN, Volume *37*

Supplemental information

Vacuolar ATPase subunit Atp6v0c transgene promotes neuroprotection and long-distance axon regeneration in injured retinal ganglion neurons

Anja Kearney, Agnieszka Lukomska, Jacob Brady, Ashiti Damania, Mahit Gupta, and Ephraim F. Trakhtenberg

MATERIALS AND METHODS

Animal use, surgeries, intraocular injections. All animal studies were performed at the University of Connecticut Health Center with approval of the Institutional Animal Care and Use Committee and of the Institutional Biosafety Committee, and performed in accordance with the ARVO Statement for the Use of Animals in Ophthalmic and Visual Research. Mice were housed in the animal facility with a 12-h light/12-h dark cycle (lights on from 7:00 AM to 7:00 PM) and a maximum of five adult mice per cage. Food and water were available ad libitum. The study used wild-type 129S1/SvImJ (JAX strain 002448) mice, as well as Thy1-YFP reporter mice (which also independently co-express Cre^{ERT2}; JAX strain 012708) transferred to 129S1/SvImJ background. In these transgenic reporter mice, within the retina, YFP labels the RGCs[1]. Optic nerve surgeries and intravitreal injections, were carried out on mice of both sexes 8-12 weeks of age (average body weight 20-26 g) under general anesthesia, as described previously[2,3]. For histological analysis, mice were euthanized using CO2 and cervical dislocation. The viruses included AAV2 vectors expressing Atp6v0c (ORF of ENSMUST00000024932), anti-Pten shRNAs (target sequences: 5′-GCAGAAACAAAAGGAGATATCA-3′, 5′-GATGATGTTTGAAACTATTCCA-3′, 5′-GTAGAGTTCTTCCACAAACAGA-3′, and 5′-GATGAAGATCAGCATTCACAAA-3′), anti-Klf9 shRNAs (target sequences: 5′-GGAGGCGCTGCCGGTTACGTA-3′, 5′-TGGCTGCCCAGTGTCTGGTTT-3′, 5′-CGGGGGACACCTGGAAGGATT-3′, and 5′-GCAAATAAATGCTTTTGGTAC-3′), and mCherry alone for control (titers ~1×10^{12} GC/mL; VectorBuilder, Inc.). Vectors expressing anti-Pten or anti-Klf9 shRNAs, also co-expressed an mCherry reporter. Atp6v0c and mCherry ORFs had an *N*-terminally fused myc-tag reporter. Viruses (2 µl per eye) were injected intravitreally, avoiding injury to the lens, in 8-week-old mice, which were randomly assigned to experimental or control conditions, 2 weeks prior to ONC surgery. This lead time allowed for sufficient transduction and expression of the transgenes in RGCs at the time of ONC. Transduction efficiency was approximately 30%, which is comparable to prior reports that also used AAV2 to target the RGCs[3]. Cholera toxin subunit B (CTB) conjugated to Alexa Fluor 488 dye (C34775, ThermoFisher Scientific) was injected (1% in 3 µl PBS) intravitreally one day prior to sacrifice, at 2 weeks after ONC, in order to visualize the regenerating axons or their absence.

Tissue processing and immunostaining. Standard histological procedures were used, as described previously[2,3]. Briefly, anesthetized mice were transcardially perfused with isotonic saline followed by 4% paraformaldehyde (PFA), the eyes and the optic nerves were dissected, the cornea was punctured, and the tissues were postfixed 2 hours. The retinas dissected-out for flat-mounts or horizontal flat-sections, the whole eyes for sagittal cross-sections, and the optic nerves for longitudinal sections, were washed in PBS and transferred to 30% sucrose overnight at 4 °C. The optic nerves were then embedded in OCT Tissue Tek Medium (Sakura Finetek), frozen, cryosectioned longitudinally at 14 μm, and then mounted for imaging on coated glass slides. For analyzing RGC survival, resected (into PBS at 4 °C) free-floating retinas were immunostained in 24-well plate wells and, after making 4 symmetrical slits, flat-mounted on coated glass slides for imaging. For analyzing Atp6v0c transgene expression in AAV2-transduced RGCs, the flattened retinas were embedded in OCT Tissue Tek Medium (Sakura Finetek), frozen, and cryosectioned at 14 μm horizontality to capture the ganglion cell layer (GCL) of the retina, and then immunostained and mounted on coated glass slides for imaging using a confocal microscope (see below). For Atp6v0c immunostaining, the whole eyes were embedded in OCT Tissue Tek Medium (Sakura Finetek), frozen, and cryosectioned at 14 μm sagittally (capturing the GCL in the cross-sections), and then immunostained and mounted on coated glass slides for imaging using a confocal microscope (see below). For immunostaining, the tissues were blocked with appropriate sera, incubated overnight at 4 °C with primary antibodies, Atp6v0c (1:100; rabbit polyclonal, PA5116676 Thermo Fisher Scientific), βIII-Tubulin (1:500; rabbit polyclonal, Ab18207 Abcam), and Myc (1:400; mouse monoclonal, SC-40 SCBT), then washed 3 times, incubated with appropriate fluorescent dye-conjugated secondary antibodies (1:500; IgG H+L or IgG 2a Alexa Fluor, Thermo Fisher Scientific) overnight at 4 °C, washed 3 times again, and mounted for imaging.

Quantification of regenerated axons and RGC survival. To visualize the regenerating axons or their absence after treatments, axonal tracer (Alexa Fluor 488-conjugated CTB 1% in 3 μl PBS) was intravitreally injected one day before animals were euthanized 2 weeks following ONC. Longitudinal sections of the optic nerve were examined for possible axon sparing. No spared axons were found in control, and no evidence of axon sparing was found in experimental conditions (i.e., at 2 weeks after injury, no axons were found at the most distal from the injury region of the optic nerve). Regenerated axons (defined as continuous fibers, which are absent in

controls and are discernible from background puncta and artefactual structures) were counted manually using a fluorescent microscope (40x/1.2 C-Apochromat W; AxioObserver.Z1, Zeiss) in at least 4 longitudinal sections per optic nerve at various distances from the injury site (identified by the abrupt disruption of axonal density approximately 1 mm from the optic nerve head, as marked by a rhombus in Fig. 3), and these values were used to estimate the total number of regenerating axons per nerve, as described[2,3]. For representative images, serial fields of view along the longitudinal optic nerve tissue section were imaged as above; *z*-stacks with 5 planes at 0.5 μm intervals were deconvoluted, merged, and stitched (ZEN software, Zeiss). Then, processed images of 3 tissue sections from the same optic nerve were superimposed over each other and merged using Photoshop CS6 (Adobe), shown as representative images. RGC survival was quantified in retinal flatmounts' GCL as described[2,3], by immunostaining with an antibody to βIII-Tubulin (neuronal marker), taking advantage of the selective expression (within the retina) of βIII-tubulin in RGCs. ImageJ software Cell Counter Plugin was used to count βIII-Tubulin positive cells from images acquired (using a fluorescent microscope, 20x LD; Zeiss, AxioObserver.Z1, Zeiss) at 1 mm and at 2 mm from the optic nerve head in four directions of the GCL, then averaged to estimate overall RGC survival per mm^2 of the retina. For analyzing RGC transduction efficiency, the horizontality cryosectioned flattened retinas' GCL immunostained for βIII-Tubulin and Myc reporter were imaged along with mCherry reporter (where applicable) using confocal microscopy (63x Oil; LSM800, Zeiss), and proportions of βIII-Tubulin+/reporter+ of total βIII-Tubulin+ RGCs were quantified; representative images were sampled randomly. For quantifications and representative images of Atp6v0c immunostained RGCs, Thy1-YFP mice's retinal GCL regions randomly sampled from the sagittally cryosectioned retinas' immunostained for Atp6v0c were imaged, along with the YFP RGC marker, using a confocal microscope (63x objective; LSM800, Zeiss). Atp6v0c immunofluorescence signal intensity was measured in the YFP+ RGCs located in the GCL, using ZEN software (Zeiss) measurements tools for average pixel intensity. Quantifications were performed on individual RGCs present in the 63x image field of view GCL, in 4 eye/retinal cross-sections per condition. Investigators performing the surgeries and quantifications were masked to the group identity by another researcher until the end of the experiment.

Statistical analyses. All tissue processing, quantification, and data analysis were done masked throughout the study. Sample sizes were based on accepted standards in the literature and our prior experiences. Sample size

represents total number of biological replicates in each condition. All experiments included appropriate controls. No cases were excluded in our data analysis, although a few animals that developed a cataract in the injured eye were excluded from the study, and their tissues were not processed. The data are presented as means ± SEM, and was analyzed (as specified in the applicable Figure legends) by independent samples *t*-test (2-tailed) or by ANOVA with or without Repeated Measures and a posthoc LSD test (SPSS). All differences were considered significant at $p < 0.05$.

RGC scRNA-seq and bulk-mRNA-seq datasets. ScRNA-seq was performed as we described previously[3], briefly: uninjured and injured (2 weeks post-ONC) RGCs from 12-week old mice of both sexes were Thy1-immunopanned from single cell retinal suspension (10 retinas per batch), after immunopanning depletion of macrophages and amacrine cells. Cells were resuspended in DPBS with 0.04% BSA, and immediately processed as follows. Cell count and viability were determined using trypan blue on a Countess FL II, and 6,000 cells per batch were loaded for capture onto the Chromium System using the v2 single cell reagent kit (10X Genomics). Following capture and lysis, cDNA was synthesized and amplified (12 cycles) as per manufacturer's protocol (10X Genomics). The amplified cDNA from each channel of the Chromium System was used to construct an Illumina sequencing library and sequenced on HiSeq 4000 with 150 cycle sequencing. Illumina basecall files (*.bcl) were converted to FASTQs using CellRanger v1.3, which uses bcl2fastq v2.17.1.14. FASTQ files were then aligned to mm39 mouse reference genome and transcriptome using the CellRanger v1.3 software pipeline with default parameters, which demultiplexes the samples and generates a gene versus cell expression matrix based on the barcodes and assigned unique molecular identifiers (UMIs) that enable determining the individual cells from which each RNA molecule originated. For determining gene expression, normalization of the raw counts was performed using Seurat v5.2.1's NormalizeData function, which divides the feature counts by the number of counts per each cell and then applies natural log transformation, resulting in normalized expression (*NE*) values. Individual samples were batch adjusted and integrated using Seurat's FindVariableFeatures, SelectIntegrationFeatures, FindIntegrationAnchors, and IntegratedData functions. RGC cell identity was confirmed based on co-expression of pan-RGC markers (Rbpms, Slc17a6, Sncg, and Tubb3). A total of 1683 RGCs from uninjured and 1375 RGCs from injured retinas that passed quality control (QC) were selected. QC filters/thresholds included the following criteria per cell: a maximum threshold of 20% mitochondrial genes

expressed in the transcriptome, a minimum of 500 genes, and a maximum of 150,000 UMIs (to mitigate the presence of cell doublets). Uninjured and injured datasets were then merged and normalized using Seurat's merge and NormalizeData functions. Violin plots of normalized gene expression were generated using Seurat's VlnPlot function, and overlayed categorical scatter (violin point) plots were generated using ggbeeswarm. Boxplots for median relative counts of normalized expression (not-logged, with median ± interquartile range error bars) of V-ATPase Vo subunit genes in the uninjured and injured RGCs were generated using Seurat's NormalizeData function (with Relative Counts normalization and a scaling factor of 1000) and visualized using ggplot2. For bulk-mRNA-seq, adult uninjured RGCs transduced with AAV2 (~30% transduction efficiency) expressing mCherry were Thy1-immunopanned from single cell retinal suspension (from 10 retinas after immunopanning depletion of macrophages) and FACS'ed for mCherry+ cells (as we described previously[3]). Approximately, 5,000 RGCs were collected by FACS and RNA was isolated immediately using the Zymo QuickRNA microprep kit. Total RNA with RNA Integrity Number (RIN) ≥ 9 (by Bioanalyzer 2100 using the Nano 6000 kit, Agilent) was extracted using Direct-zol RNA MiniPrep kit (R2050, Zymo Research). cDNA libraries were prepared using polyA-selected RNA (TruSeq RNA Library Prep Kit, Illumina). Paired reads were sequenced in a DNA-strand-specific manner, 100 bp from each end on HiSeq 2000 Sequencer (Illumina), passed QC filters, mapped to the mm39 genome and transcriptome by Hisat2, and gene expression was analyzed by Cufflinks (following a pipeline that we previously published). Transcript isoforms expressed > 1 FPKM in both replicates were visualized using IGV browser.

Data availability. The scRNA-seq dataset from adult uninjured and injured RGCs that we generated for these studies is available through the NCBI GEO accession GSE325128. Adult uninjured RGC bulk-mRNA-seq raw reads and processed data for expression of transcripts isoforms from the Atp6v0c gene locus analyzed in this study are available through the NCBI GEO under accession number GSE252517 (which we generated previously).

METHODS FOR FIGURE S1

Gene-concept network plot analysis was performed using the methods we described previously[3-5]. Briefly, genes differentially expressed (logfc.threshold = 0.4, min.pct = 0.5) in adult uninjured vs injured RGCs were identified using Seurat's FindMarkers function[6,7]. Those expressed > 0.1 *NE* in the higher expressing condition were analyzed using the R package clusterProfiler, using all genes expressed in injured RGCs as background (*NE* > 0). False discovery rate (FDR) was used for multiple testing correction, and a minimum *p*-value of 0.05 was set as cutoff for significance of GO terms enrichment. GO:BP terms were rank ordered by decreasing significance (FDR adjusted *p*-value). Fold-enrichment was calculated as the DEG ratio divided by the background ratio[8]. Significantly enriched GO:BP pathways (*p*-value < 0.05) containing Atp6v0c gene were plotted in a Gene-Concept Network Plot using the clusterProfiler and enrichplot R packages[9].

Validation of Atp6v0c transgene expression by scRNA-seq. RGC scRNA-seq is detailed in the main text Materials And Methods, and was performed similarly on the RGCs treated with AAV2-Atp6v0c. Briefly, at 2 weeks post-ONC, CTB-488 stereotaxically injected into the distal optic nerve uptaken by the regenerated axons and retrogradely transported to the soma of RGCs in the retina. The next day, the responding RGCs FACS-isolated and processed by scRNA-seq, as described in the main text Materials And Methods, with the following additional steps. AAV2 vector transgene with UTRs was custom-added to the reference genome and transcriptome GTF. Reads mapping to the AAV2 vector transgene UTRs were detected only in the AAV2-Atp6v0c-treated condition, confirming transgene expression in the responding RGCs. For determining Atp6v0c gene *NE*, counts of the reads that mapped non-redundantly (i.e., reads that mapped to both were counted only ones) to the endogenous and transgene Atp6v0c were combined. More detailed methods for these procedures are described in our previous publications[3,10-12].

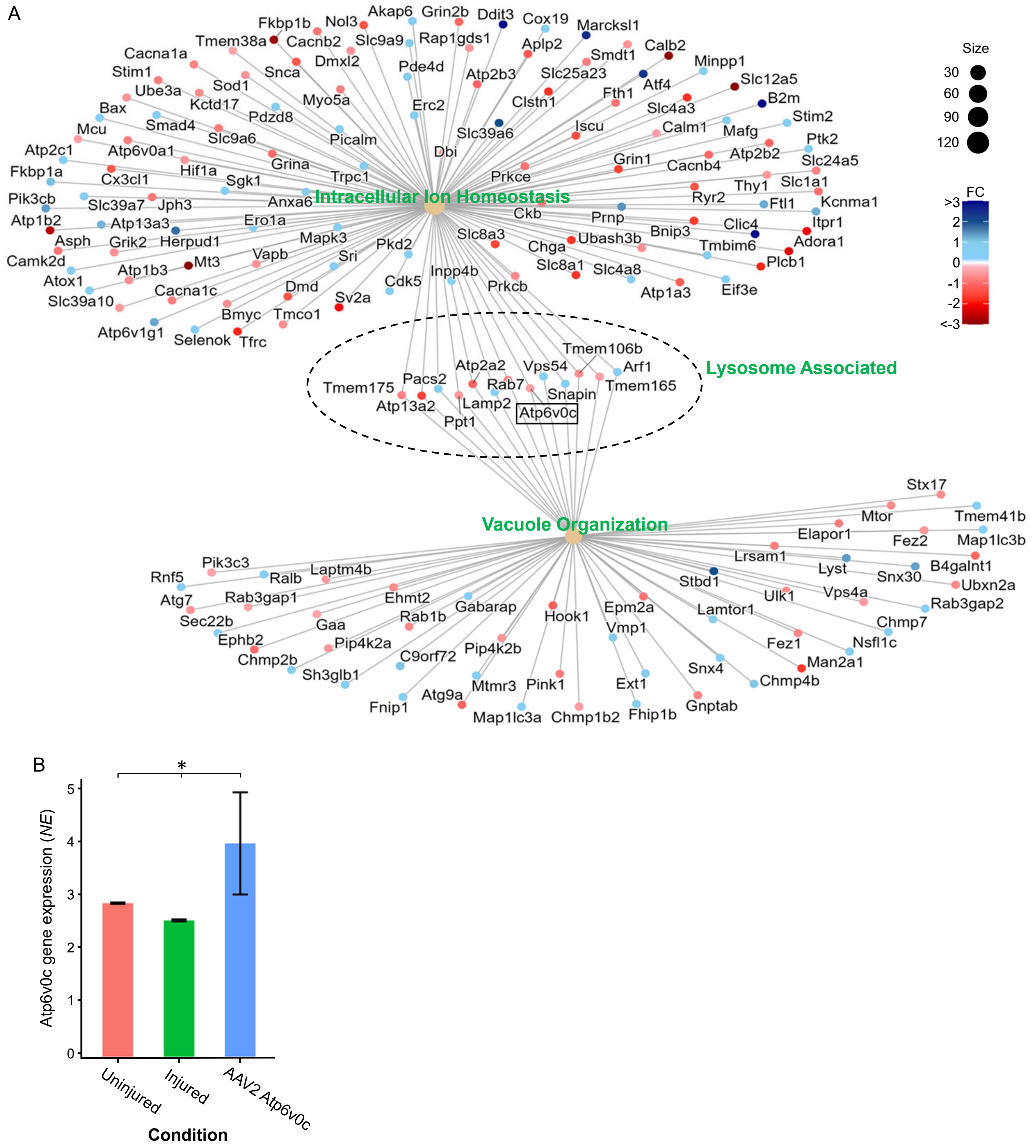

Figure S1. (**A**) Gene-Concept Network Plot of injury-regulated genes associated with GO:BP terms containing Atp6v0c (outlined with a solid line rectangular). GO terms annotated in green font represented by orange circles (*i.e.*, nodes), with circle size (per scale on the side) indicating the number of genes within that node. Color-coded scale bar indicates the log2 fold-change (FC) in expression between adult uninjured and injured RGCs. GO terms' Fold Enrichment was 1.45 (p < 0.001) for Intracellular Ion Homeostasis and 1.66 (p < 0.001) for Vacuole Organization. Shared genes between these two GO terms, outlined by a dashed oval, are manually curated as Lysosome Associated based on literature review. (**B**) Average gene expression of Atp6v0c is significantly increased in AAV2-Atp6v0c-treated injured RGCs relative to both uninjured and injured-control RGCs. scRNA-seq reads mapping to the AAV2 vector transgene UTRs were detected only in the AAV2-Atp6v0c-treated condition, in which endogenous and transgene Atp6v0c were combined for determining *NE* value. Mean (SEM) of *NE* values (log-normalized) are shown; significant differences (p < 0.05, indicated by an asterisk) by ANOVA with posthoc LSD.

SUPPLEMENTAL REFERENCES

1. Hass DT, Barnstable CJ. Mitochondrial Uncoupling Protein 2 Knock-out Promotes Mitophagy to Decrease Retinal Ganglion Cell Death in a Mouse Model of Glaucoma. *J Neurosci*. May 01 2019;39(18):3582-3596. doi:10.1523/JNEUROSCI.2702-18.2019

2. de Lima S, Koriyama Y, Kurimoto T, Oliveira JT, Yin Y, Li Y, Gilbert H, Fagiolini N, Martinez AMB, and Benowitz L. Full-length axon regeneration in the adult mouse optic nerve and partial recovery of simple visual behaviors. *Proc Natl Acad Sci U S A*. Jun 2012;109(23):9149-54. doi:10.1073/pnas.1119449109

3. Rheaume BA, Xing J, Lukomska A, Theune WC, Damania A, Sjogren G, and Trakhtenberg EF. Pten inhibition dedifferentiates long-distance axon-regenerating intrinsically photosensitive retinal ganglion cells and upregulates mitochondria-associated Dynlt1a and Lars2. *Development*. Apr 15 2023;150(8)doi:10.1242/dev.201644

4. Xing J, Theune WC, Lukomska A, Frost MP, Damania A, and Trakhtenberg EF. Experimental upregulation of developmentally downregulated ribosomal protein large subunits 7 and 7A promotes axon regeneration after injury in vivo. *Exp Neurol*. Aug 24 2023:114510. doi:10.1016/j.expneurol.2023.114510

5. Lukomska A, Frost MP, Theune WC, Xing J, Gupta M, and Trakhtenberg EF. Nfe2l3 promotes neuroprotection and long-distance axon regeneration after injury in vivo. *Exp Neurol*. Feb 21 2024:114741. doi:10.1016/j.expneurol.2024.114741

6. Stuart T, Butler A, Hoffman P, Hafemeister C, Papalexi E, Mauck WM 3rd, Hao Y, Stoeckius N, Smibert P, and Satija R. Comprehensive Integration of Single-Cell Data. *Cell*. 06 2019;177(7):1888-1902.e21. doi:10.1016/j.cell.2019.05.031

7. Hao Y, Hao S, Andersen-Nissen E, Mauck WM 3rd, Zheng S, Butler A, Lee MJ, Wilk AJ, Darby C, Zager M, et al. Integrated analysis of multimodal single-cell data. *Cell*. 06 24 2021;184(13):3573-3587.e29. doi:10.1016/j.cell.2021.04.048

8. Mi H, Muruganujan A, Casagrande JT, Thomas PD. Large-scale gene function analysis with the PANTHER classification system. *Nat Protoc*. Aug 2013;8(8):1551-66. doi:10.1038/nprot.2013.092

9. Wu T, Hu E, Xu S, Chen M, Guo P, Dai Z, Feng T, Zhou L, Tang W, Zhan L, et al. clusterProfiler 4.0: A universal enrichment tool for interpreting omics data. *Innovation (Camb)*. Aug 28 2021;2(3):100141. doi:10.1016/j.xinn.2021.100141

10. Theune WC, Frost MP, Trakhtenberg EF. Transcriptomic profiling of retinal cells reveals a subpopulation of microglia/macrophages expressing Rbpms marker of retinal ganglion cells (RGCs) that confound identification of RGCs. *Brain Res*. Jul 15 2023;1811:148377. doi:10.1016/j.brainres.2023.148377

11. Trakhtenberg EF. Single-cell transcriptomics-enabled advances in experimental optic nerve axon regeneration research. In: Bhattacharya SK, ed. *Proteomics, Multi-Omics and Systems Biology in Optic Nerve Regeneration*. Academic Press; 2025:207-223:chap 14.

12. Rheaume BA, Jereen A, Bolisetty M, Sajid MS, Yang Y, Renna K, Sun L, Robson P, and Trakhtenberg EF. Single cell transcriptome profiling of retinal ganglion cells identifies cellular subtypes. *Nat Commun*. Jul 2018;9(1):2759. doi:10.1038/s41467-018-05134-3
